# The Bifactor Model Fits Better Than the Higher-Order Model in More Than 90% of Comparisons for Mental Abilities Test Batteries

**DOI:** 10.3390/jintelligence5030027

**Published:** 2017-07-11

**Authors:** Jeffrey Cucina, Kevin Byle

**Affiliations:** U.S. Customs and Border Protection, 1400 L Street, NW, Washington, DC 20229-1145, USA; kevin.a.byle@cbp.dhs.gov

**Keywords:** intelligence, mental-abilities, factor analysis, bifactor, higher-order

## Abstract

The factor structure of mental abilities has most often been depicted using a higher-order model. Under this model, general mental ability (*g*) is placed at the top of a pyramid, with “loading” arrows going from it to the other factors of intelligence, which in turn go to subtest scores. In contrast, under the bifactor model (also known as the nested factors/direct hierarchical model), each subtest score has its own direct loading on *g*; the non-*g* factors (e.g., the broad abilities) do not mediate the relationships of the subtest scores with *g*. Here we summarized past research that compared the fit of higher-order and bifactor models using confirmatory factor analysis (CFA). We also analyzed additional archival datasets to compare the fit of the two models. Using a total database consisting of 31 test batteries, 58 datasets, and 1,712,509 test takers, we found stronger support for a bifactor model of *g* than for the traditional higher-order model. Across 166 comparisons, the bifactor model had median increases of 0.076 for the Comparative Fit Index (CFI), 0.083 for the Tucker-Lewis Index (TLI), and 0.078 for the Normed Fit Index (NFI) and decreases of 0.028 for the root mean square error of approximation (RMSEA) and 1343 for the Akaike Information Criterion (AIC). Consequently, researchers should consider using bifactor models when conducting CFAs. The bifactor model also makes the unique contributions of *g* and the broad abilities to subtest scores more salient to test users.

## 1. Introduction

Examinations of the factor structure of mental ability test batteries are a common research topic in intelligence and assessment. Indeed, the internal structure of a test is an important aspect of the evaluation of validity and reliability according to the “Standards for Educational and Psychological Testing” (American Educational Research Association [1]). Researchers analyzing the factor structure of mental ability test batteries must make decisions about how to model general mental ability (i.e., *g*). Traditionally, *g* has been conceptualized using a higher-order method; however, the bifactor model (also known as the nested factors/direct-hierarchical model^1^) has experienced increasing use recently.

The purpose of this paper is to compare the fit for bifactor and higher-order models using multiple mental ability test batteries and datasets from various disciplines in psychology (e.g., clinical, educational, and industrial). Two competing hypotheses for the modeling of *g* are tested in this paper; the higher-order model and the bifactor model. This paper describes the higher-order and bifactor models and then empirically compares the fits of these models using multiple test batteries and datasets. Our empirical comparison summarizes the existing literature and adds multiple new sources of data to provide the most comprehensive comparison of the higher-order and bifactor methods to date. We begin with an explanation of the two models and a review of past research. We then present the results of the empirical comparisons of the models and provide interpretative comments on our findings in the Discussion section.

### 1.1. Higher-Order Model

**Figure 1:**
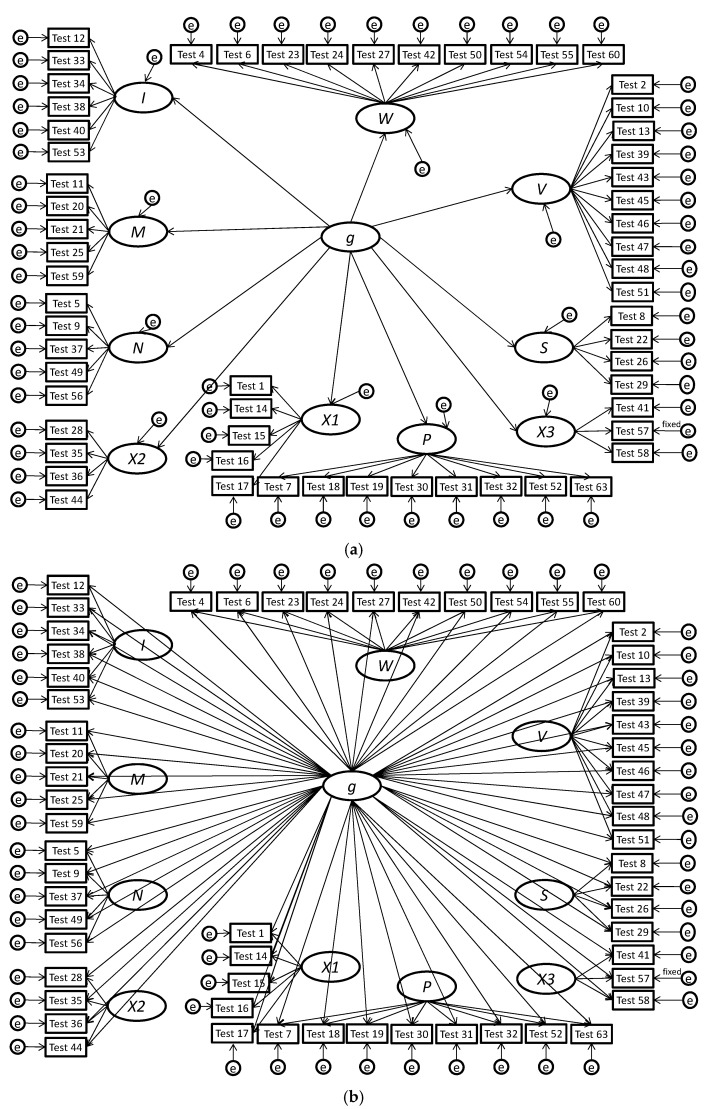
(**a**) This figure shows the higher-order model for the Thurstone and Thurstone [8] test battery. For the sake of clarity, only primary loadings are depicted. The model that only included primary loadings had an RMSEA of 0.063, an AIC of 6696, and a *χ*^2^ of 6438 (*df* = 1,701, *p* < 0.001). Due to a Heywood case, the error variance for Test 57 was fixed to the value implied by its reliability; (**b**) This figure presents the corresponding bifactor model for the Thurstone and Thurstone [8] test battery. This model, which only had primary loadings, had an RMSEA of 0.059, an AIC of 6093, and a *χ*^2^ of 5737 (*df* = 1652, *p* < 0.001). A *Δχ*^2^ of 701 (*df* = 49, *p* < 0.001) indicated that it had statistically significant incremental fit over the higher-order model. Due to a Heywood case, the error variance for Test 57 was fixed to the value implied by its reliability.

**Figure 2:**
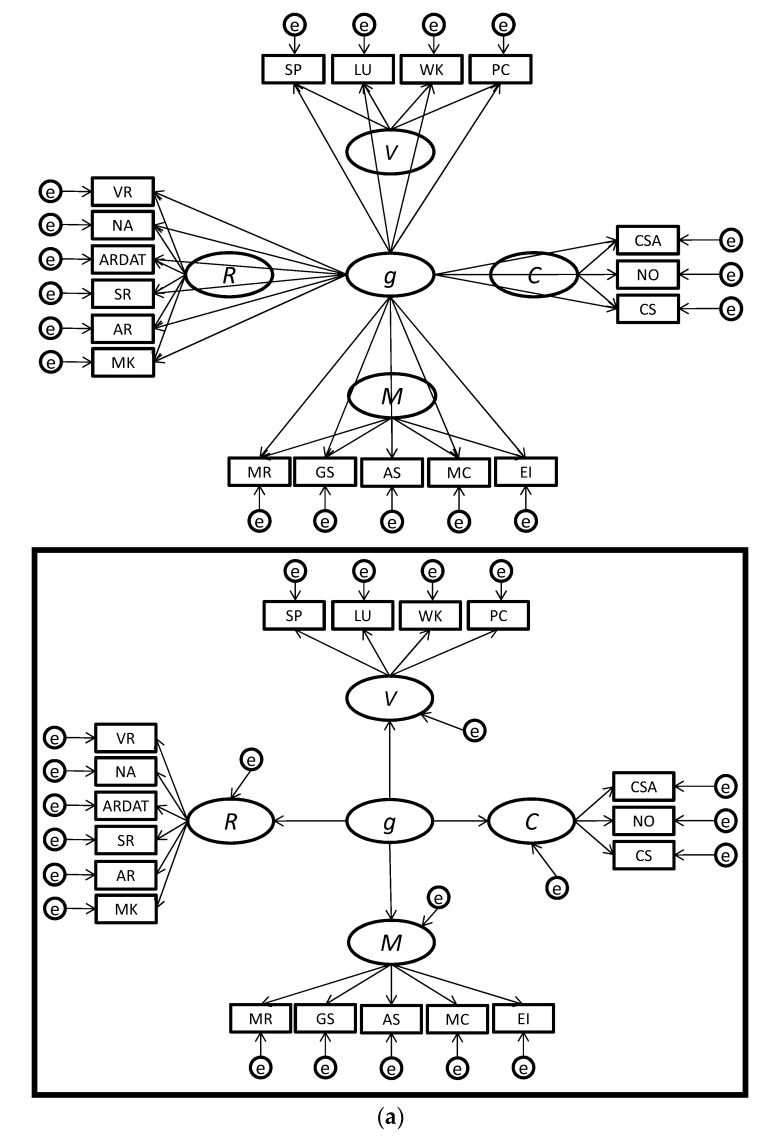

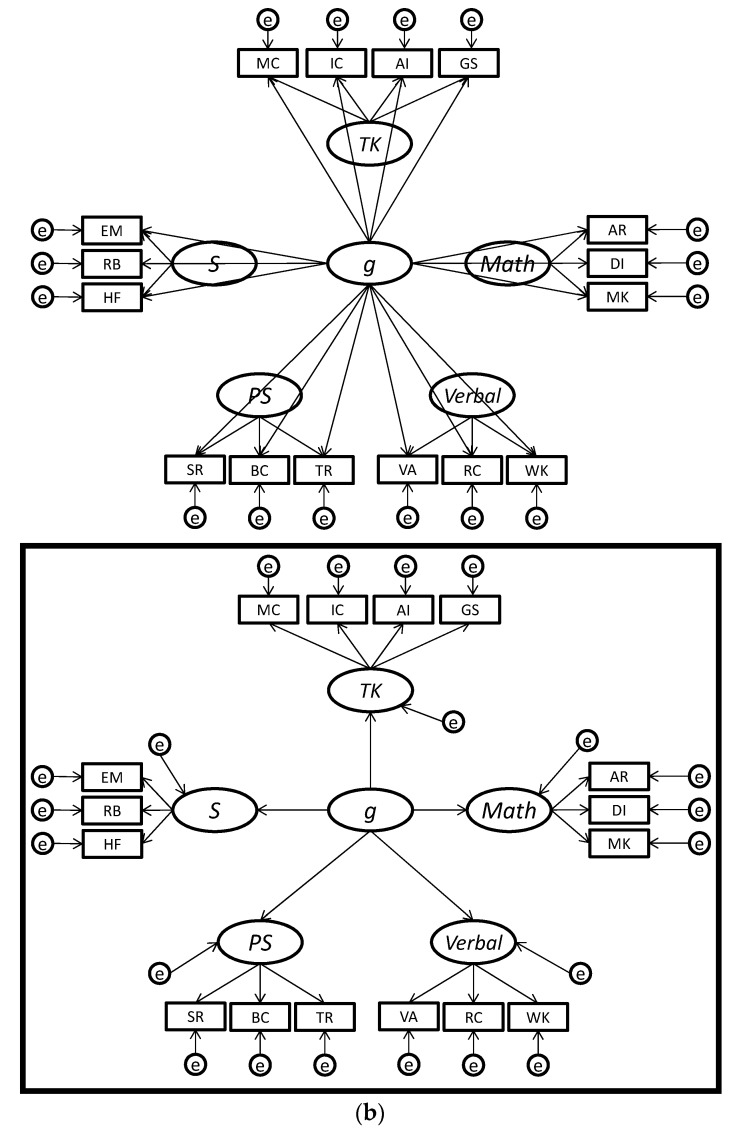
(**a**) This figure shows the bifactor model for the DAT-ASVAB test battery, with the corresponding higher-order model shown in the inset; (**b**) This figure shows the bifactor model for the AFQOT battery, with the corresponding higher-order model shown in the inset. For the sake of clarity, only primary loadings are depicted. The bifactor model that only included primary loadings had better fit statistics (Comparative Fit Index (CFI) = 0.924; Tucker-Lewis Index (TLI) = 0.897; Normed Fit Index (NFI) = 0.921; Akaike Information Criterion (AIC) = 2333) than the higher-order model (CFI = 0.900; TLI = 0.879; NFI = 0.897; AIC = 3000), and the *Δχ*^2^ of 690 (*df* = 11, *p* < 0.001) was significant.

One way to distinguish between the higher-order and bifactor models of the factor structure of mental abilities is through a comparison of path diagrams, which are shown in Figure 1a,b and Figure 2a,b. In the higher-order conceptualization of the factor structure of mental abilities, subtest scores are effect indicators (Bollen and Lennox [2]) of the broad mental abilities, which are, in turn, effect indicators of *g*. This is evident in the depiction of the higher-order model in Figure 1a and the insets of Figure 2a,b. Under a higher-order model, whether or not an individual has a high or low standing on a broad ability such as crystallized intelligence depends on (a) their level of *g*, (b) their personal investment (e.g., interest, exposure, motivation) in developing crystallized intelligence, and (c) some possible unique innate ability (or inability) in crystallized intelligence. Note that, in the higher-order model, the effects of *g* on a subtest score are “fully mediated” by the lower-level factor (e.g., crystallized intelligence). Subtest scores are connected to *g* via two paths, one that goes from *g* to the broad ability factor and another that goes from the broad ability factor to the subtest scores. For example, spelling ability subtest scores only indirectly measure *g*, and *g* does not directly cause the scores on the subtest. This means that the effects of *g* on the subtest scores are constrained by the broad factor. If the broad factor has a small correlation with *g*, then the subtest scores will also have a small correlation with *g*.

### 1.2. Bifactor Model

In the bifactor model, each subtest’s score loads directly on *g* and on the broad factors (e.g., crystallized intelligence). Thus, under the bifactor model, *g* has its own loadings with subtest scores rather than indirect loadings via the broad factors. Figure 1b and Figure 2a,b present depictions of the bifactor model. Note that, in the bifactor model, the subtest scores are effect indicators of both *g* and the broad factor. This approach allows a subtest to have a relationship with *g* that is not constrained by the broad factor.

The bifactor model of *g* was originally hypothesized by Holzinger and Swineford [3,4]. However, it did not receive widespread usage compared to the higher-order model, historically speaking. In their review, Reeve and Blacksmith [5] reported that only 22.7% of confirmatory factor analysis (CFA) studies used a bifactor model (as opposed to 57.6% for the higher-order model). Many intelligence researchers (e.g., Jensen [6]; Jensen and Weng [7]) have ambivalently discussed the bifactor model in their writings. However, a few researchers have endorsed a bifactor model of intelligence.

John Carroll, developer of the Carroll [9] three-stratum theory of mental abilities, often used the bifactor model (see Beaujean [10]). Later, Gignac [11,12,13,14] and others (e.g., Collinson, Evans, Wheeler, Brechin, Moffitt, Hill, and Muncer [15]) published a series of papers showing that the bifactor model provides better fit, leading to a resurgence in the use of this model, which was observed by Reise [16]. Nevertheless, the higher-order model remains the predominantly used approach.

### 1.3. Model Building

The bifactor and higher-order models can also be distinguished as top-down and bottom-up model building approaches (Jensen [6]). The bifactor model follows a top-down approach as the model builder begins with the most prominent factor, *g*, and allows all of the subtest scores to load onto it. The model builder then adds subsequent factors and allows certain subtest scores to load onto each of the subsequent factors. The higher-order model follows a bottom-up approach by beginning with the subtest scores and allowing each subtest score to load onto broad ability factors. The broad abilities factors then load onto *g*; sometimes a second layer of specific abilities factors is also included (per Carroll’s [9] Three-Stratum Theory).

### 1.4. Past Research and the Need for Further Research

A number of studies, which are summarized in Table 1, have reported that the bifactor model provides better fit statistics than the higher-order model. Despite this research, there has not been a summary review of the previous research comparing the higher-order and bifactor models to date. Indeed, Reynolds and Keith [17] noted that whether or not mental abilities are best viewed under a higher-order or bifactor model remained an “unresolved issue” (p. 269). Our paper addresses this gap in order to provide insight into which model provides better fit. This is accomplished by using the results of previous studies and adding new results from additional sources of data from across multiple disciplines of psychology.

In addition, recent research has called into question the superiority of the fit of the bifactor model. Using a Monte Carlo simulation, Murray and Johnson [18] made a case that the bifactor model can incorrectly lead to better fit when, in actuality, the higher-order model is the true model if unmodeled complexity (e.g., correlated residuals, cross loadings) exists in the higher-order model. They concluded that the increased model fit associated with bifactor models cannot be taken as evidence that it is the true underlying model. Gignac [19] has provided another interpretation of this conclusion. He noted that Murray and Johnson [18] did not consider proportionality constraints in their Monte Carlo simulation. Using the results of his own simulation, Gignac [19] found that violations of the proportionality constraint were correlated with the incremental fit of the bifactor model over the higher-order model. He concluded that Murray and Johnson’s [18] findings were due to the fact that adding unmodeled complexity introduced violations to the proportionality constraint. He also found that one fit statistic, TLI, in fact favored the higher-order model, as opposed to the bifactor model, when the degree of the proportionality constraint violation was low, and he indicated that fit statistics that include penalties for model complexity may incorrectly favor the higher-order model. Additionally, he suggested that use of the higher-order model requires an explanation of why there should be a proportionality constraint, whereas use of the bifactor model does not. In a follow-up article, Mansolf and Reise [20] suggested that the bifactor and higher-order models can be contrasted in terms of tetrad constraints, and they recommended three additional tests for comparing the two models. They also conducted a Monte Carlo simulation, which showed that, when there are large levels of unmodeled complexity, the BIC fit statistic slightly favors the bifactor model. However, when the level of unmodeled complexity was low or moderate, the BIC fit statistic favored the higher-order model.

In our opinion, a major issue with unmodeled complexities is the lack of conceptual grounding based on prior theoretical work (e.g., Carroll’s [9] Three Stratum Theory) or cross-validated analyses. There is something to be said for hypothesizing the factor structure of a test *a priori* rather than hypothesizing after the results are known (HARKing) via modification indices. It is possible to make enough modifications to either the higher-order or bifactor model to achieve near perfect fit. Thus, it might be best to develop an *a priori* factor structure that incorporates all possible terms that can legitimately be hypothesized (based on test content and prior theoretical work) and then compare the fit of the higher-order and bifactor models. Another approach is the Bayesian structural equation modeling that Golay, Revert, Rossier, Favez, and Lecerf [42] employed, whereby previously unmodeled cross-loadings can be included in the model, albeit with prior distributions that center around zero. Using this approach, Golay et al. [42] found that the bifactor model provided better fit than the higher-order model even when the cross-loadings were modeled. Thus, the Bayesian structural equation modeling results suggest that unmodeled complexity does not lead to erroneous support for the bifactor model.

Although they did not study unmodeled complexity, Morgan, Hodge, Wells, and Watkins [43] conducted a follow-up Monte Carlo simulation to Murray and Johnson [18] to examine bias in fit statistics. When using a four-factor model with three indicators per factor, they found that when the true underlying model was higher-order, the bifactor model was selected 64% to 72% of the time (depending on whether the Comparative Fit Index (CFI), Tucker-Lewis Index (TLI), Root Mean Square Error of Approximation (RMSEA), or Standardized Root Mean Residual (SRMR) was used) when there were 200 cases and 66% to 83% of the time when there were 800 cases. When the true underlying model was bifactor, the bifactor model was selected 85% to 97% of the time for 200 cases and 100% of the time for 800 cases. In contrast, the higher-order model was only selected 2% to 33% of the time for 200 cases and 0% to 3% of the time for 800 cases when the true underlying model was bifactor. These results do call into question the inferences that can be made from a single study that compares the bifactor and higher-order models. However, when the results from multiple datasets are accumulated and large sample sizes are used, especially if the aggregated results clearly support one model over the other, more definitive conclusions about the fit of the bifactor and higher-order models can be made. Prior research [43] did indicate that when the true model is higher-order, the bifactor model is selected between 64% and 83% of the time (depending on the fit statistic and number of cases). However, suppose that it is found that the bifactor model is supported nearly 100% of the time across multiple datasets and analyses, showing that the observed frequency of support for the bifactor model exceeds the frequency that is expected due to bias in fit statistics. This type of analysis requires the accumulation and analysis of a large number of studies. Our study aimed to address this gap in the literature by accumulating and analyzing such a database.

Another limitation of past work on this topic is that it has not often included many of the mental ability test batteries used in other areas of psychology (e.g., industrial psychology) nor the seminal large-scale ability test batteries published in the literature. This is surprising since the same types of mental ability constructs are assessed across different areas of psychology. Our study addresses this limitation by including data from multiple disciplines.

## 2. Materials and Methods

### 2.1. Selection of Datasets

The current study compares the bifactor and higher-order models using data from test batteries that were developed, used, or published by applied psychologists (including clinical, educational, and industrial), as well as published research-based studies and datasets with large numbers of subtests (i.e., at least three per factor) and subjects. Literature searches were conducted in American Psychological Association’s PsycInfo and Google Scholar by searching for relevant terms (e.g., “bifactor”, “nested factors”, “direct hierarchical model”) and examining articles that cited research articles on the bifactor model. Next, a review of the literature was conducted to identify commonly-used mental ability test batteries and seminal factor analytic studies of mental ability test batteries. When selecting test batteries for inclusion, we focused on those that (a) had a published correlation matrix, (b) had a large number of subjects (over 500), (c) had enough subtests to form mental abilities factors, (d) were not previously studied in CFA comparisons of bifactor and higher-order models, and (e) were available to us (e.g., through interlibrary loan, internet, published articles). The datasets were identified by conducting literature searches for test manuals and technical reports for prominent IQ test batteries, employment test batteries, and educational test batteries. In addition, Carroll’s [9] datasets (McGrew [44]) were reviewed for possible inclusion. Some datasets were identified through APA PsycInfo and Google Scholar searches; however, it proved difficult to identify which papers included correlation matrices (which were necessary for conducting the CFAs). We limited the datasets to those that came from normal, non-referred populations such as standardization samples, applicant and incumbent databases, test manuals, and large-sample laboratory studies.

### 2.2. Test Batteries and Datasets

We identified 12 test batteries, 17 datasets, and 16,978 cases from previously published comparisons of the two models. We identified an additional 27 batteries, 41 datasets, and 1,695,531 cases that we analyzed ourselves. In total, our database consists of 31 batteries, 58 datasets, and 1,712,509 cases (some tests overlapped the two groups). In the Supplementary Materials, we provide background information on the batteries (Table S1) and datasets (Table S2). Most of the data came from correlation matrices, means (*M*s), standard deviations (*SD*s), and reliabilities published in journals, technical reports, monographs, and test manuals. The Project Talent datasets and the two NLSY-ASVAB datasets were obtained from published raw data that are publicly available. We used the factor structures proposed by the authors of each dataset as well as linkages provided by McGrew and Flanagan [45]. When this information was unavailable, we rationally linked the content of each subtest to the Three-Stratum Theory as described by Carroll [9] and McGrew [46]. Note that, in some cases, multiple factor structures have been proposed for the different batteries. In most cases, we only present the results for the best fitting models. However, we do provide results for additional models in certain instances (e.g., when both had good fit or when the test publisher provided a proposed factor structure in addition to one based on Carroll’s model). In some cases, the *M*s and *SD*s were not reported in the publications with the correlation matrices. Whenever these values were not obtainable, *T*-scores were used (i.e., the *M* was set to 50 and the *SD* was set to 10). Finally, in instances where the upper diagonal of the correlation matrix was presented and differed from the value in the lower diagonal (due to typographical errors), the lower diagonal value was used in all analyses. This mainly happened in a few instances with the Kettner [47] report.

### 2.3. Data Analysis Approach/Philosophy

All analyses were conducted using AMOS 4.0 with maximum-likelihood estimation. In most cases, an SPSS correlation matrix .sav file (containing intercorrelations, *M*s, *SD*s, and the sample size) was used as the dataset for AMOS, with the exception of three datasets (i.e., the NLSY 1979 and 1997 studies and the ProjectTALENT study) for which we had access to the raw data. Since (a) the goal of the study was to compare the higher-order and bifactor models (not to identify the best possible fitting model for each battery) and (b) model modifications capitalize on chance and are no longer confirmatory, no model modifications were conducted in the main analyses, (i.e., all of the models were specified *a priori*). Since our focus was on the modeling of *g* using the higher-order and bifactor models, we did not study the correlated factors model as it explicitly excludes the modeling of *g*. Additionally, given the prominence of *g* in mental ability subtest scores, in the prediction of performance (Ree and Earles [48]; Ree, Earles, and Teachout [49]; Cucina, Peyton, Su, and Byle [50]), and in diagnostic usefulness (Glutting, Watkins, Konold, and McDermott [51]), we felt that it was important to explicitly include *g* in our CFA models. Further, we focused on comparisons of the bifactor and higher-order models rather than examining absolute fit. Along these lines, we developed factor models *a priori* by either using factor structures that were published previously in the literature or rationally linking subtests to factors in the Three-Stratum Theory [9]. These factor structures were the basis for the higher-order and bifactor models that we tested. In no case did we make substantive modifications to the factor structures after running the CFAs. Thus, the models were almost entirely specified *a priori*, and empirical model building did not occur. That said, there were instances in which the models did not run successfully (e.g., Heywood cases occurred). In these situations, we made slight modifications to the models so that it would run. We made the changes not only to the model that yielded the issue (e.g., the higher-order model), but also to the other model (e.g., the bifactor model) so that we could compare them as nested models. However, we also analyzed an unmodified baseline version of the other model (e.g., the bifactor model). When Heywood cases occurred, they were resolved by fixing factor variances to 0.01 and uniquenesses to their measurement error variance. As suggested above, if the Heywood case occurred on a variable for one model (e.g., the higher-order model) but not the other model (e.g., the bifactor model), then we fixed the factor variance or error variance in both models to a low value (i.e., 0.01). This was done to ensure that the models would be nested and to provide a more direct comparison of the bifactor and higher-order models. However, we also provided the results for CFAs conducted on the unmodified models (e.g., if the bifactor model had a Heywood case, we also provided the results for the higher-order model without any fixed variances) for those analyses that did not use correlated errors, fixed variances, or dropped factors to handle situations when there were only two indicators for a factor (as described below).

In order to fit a bifactor model, at least three subtests must have loadings on each factor. Some batteries included one or more factors that only had loadings with one or two indicators (i.e., observed variables for subtest scores), which presents identification issues. When only one indicator was present, we simply ignored the factor and only included a path from *g* to the indicator. When two indicators were present (e.g., indicators A and B), we resolved the identification issue by (a) creating a “mixed” model, wherein the two indicator factor was modeled as higher-order and the remaining factors were modeled using a bifactor approach, (b) dropping the factor from our model but allowing the uniqueness terms for the factor’s indicators (i.e., A and B) to covary via “correlated errors”, (c) “dropping” the factor and not allowing the uniquenesses for A and B to covary, and (d) “fixing” the unique variances for A and B to the value implied by their reliabilities. All approaches were used for each involved test battery, thus there are four sets of results. These approaches were applied to both the higher-order and the bifactor models, allowing for a direct and fair comparison for the fit of the two models. To allow for consistency in our methodology across batteries and samples, we decided to use the four approaches in all situations rather than choosing different approaches (e.g., the best fitting approach) for each battery and sample.

Three approaches were used to compare the fit statistics. First, Yung et al. [52] provided a proof showing that the higher-order model is nested within the bifactor model and that the two models can be compared using the *Δχ*^2^ test^2^. Second, model comparisons were conducted using the Akaike Information Criterion (AIC). Third, we compared the Normed Fit Index (NFI), Comparative Fit Index (CFI), Tucker-Lewis Index (TLI), and Root Mean Square Error of Approximation (RMSEA) of the models. We focused our attention on CFI, TLI, and NFI for models with fewer variables and on RMSEA for models with more variables. In all cases, we compared the bifactor model to its corresponding higher-order model.

## 3. Results

Fit statistics for the best fitting models for each dataset are available in the Supplementary Materials. The left half of Table S3 presents the fit statistics for the higher-order models, the right half presents those for the bifactor models, and *Δχ*^2^ is presented in the middle; tests for which the bifactor model had significant incremental fit are in blue, and those for which the higher-order model had better fit are in red. For each fit index, we highlight the better fitting model using bold font. A quick scan of Table S3 shows that the bifactor model provided better fit in nearly every analysis.

Three types of comparisons were used to compare the fit of the bifactor and higher-order models. First, *Δχ*^2^ comparisons were conducted to evaluate the statistical significance of incremental fit. Second, we tallied the number of times the bifactor model had nominally better fit statistics than the higher-order model (and vice versa). Third, we averaged the fit statistics across all models (in addition to computing the median and *SD*). We were unable to locate any prior research or published guidance on meta-analyzing or averaging fit statistics from multiple test batteries. Thus, this analysis was conducted for illustrative purposes.

Of the 166 *Δχ*^2^ comparisons, the bifactor model increased the fit 164 times, indicating that the bifactor provides fit that is better than the higher-order model in terms of statistical significance. Regarding the fit statistics, across all of the analyses, the CFIs were higher for the bifactor model than for the higher-order model in 165 out of the 166 analyses and equal in one analysis. Out of the 166 analyses, the bifactor model had better fit than the higher-order model 156 times for TLI (three were equal), 166 times for NFI, 156 times for RMSEA (three were equal), and 165 times for AIC. The median CFI for the bifactor model (median = 0.922; *M* = 0.885, *SD* = 0.128) was higher than that for the higher-order model (median = 0.846; *M* = 0.801; *SD* = 0.191). Similar results were observed for TLI (bifactor: median = 0.880, *M* = 0.842, *SD* = 0.198; higher-order: median = 0.797, *M* = 0.737, *SD* = 0.364), and NFI (bifactor: median = 0.912, *M* = 0.871, *SD* = 0.150; higher-order: median = 0.834, *M* = 0.772, *SD* = 0.290). The bifactor also had lower values than the higher-order model for RMSEA (bifactor: median = 0.085, *M* = 0.093, *SD* = 0.044; higher-order: median = 0.113, *M* = 0.120, *SD* = 0.065) and AIC (bifactor: median = 1534, *M* = 25,007, *SD* = 90,262; higher-order: median = 2877, *M* = 35,653, *SD* = 137,978). (Recall that lower values for RMSEA and AIC indicate better fit).

As mentioned earlier, Morgan et al. [43] conducted a Monte Carlo simulation examining bias in statistical tests comparing the bifactor and higher-order models. We conducted a rough *post hoc* analysis to see if the frequency of the bifactor model fitting better (using CFI, TLI, and RMSEA) than the higher-order model was different from that expected based on Morgan et al.’s [43] results for situations in which the bifactor or the higher-order model was the true underlying model. For example, in our study, we found that the bifactor model had higher CFIs than the higher-order model in 165 of the 166 analyses. Morgan et al. [43] found that, when the bifactor model was the true model, it had CFI values that were higher than those for the higher-order model between 91% and 100% of the time. This means that, based on their results, it is expected that the bifactor model would have better fit for between 151 and 166 of the analyses. Using *χ*^2^ tests, we compared our observed results (e.g., 165 for CFI) to the expected results (e.g., 151 to 166 for CFI) based on Morgan et al.’s [43] simulation. Our observed results did not differ from the expected values when a bifactor model was fit to data that had a true underlying bifactor model. As shown in Table 2, all *p*-values were non-significant and all *χ*^2^ values were 1.6 or less (*df* = 1). We also compared our observed results to the expected values when a bifactor model was fit to data that had an underlying higher-order model. For CFI, all *p*-values were 0.022 or less and the *χ*^2^ values ranged from 5.3 to 16.9. For TLI and RMSEA, all *p*-values were 0.001 or less. The TLI *χ*^2^ values ranged from 10.1 to 19.2; the RMSEAs ranged from 18.2 to 23.6. Thus, although comparisons involving the bifactor and higher-order models may be biased in favor of the former, this analysis suggests that our results exceed the level of bias and that the bifactor model appears to have better fit. A limitation of this analysis is that Morgan et al. used different factor models than those that were contained in our study. Further, it should be noted that Morgan et al.’s [43] study did not incorporate unmodeled complexity (e.g., correlated errors, cross-loadings). Thus, we turn to two additional analyses suggested by Mansolf and Reise [20].

### 3.1. Additional Analyses

Recently, other proposals for comparing the higher-order and bifactor models have emerged, including the use of modification indices, creating pure factor clusters, and assessing each case’s contribution to chi-square (Mansolf and Reise [20]; Yang, Spirtes, Scheines, Reise, and Mansolf [53]). We examined the use of modification indices with the three datasets for which we had access to the raw data: the 1979 and 1997 NLSY ASVAB and the ProjectTALENT datasets (separate analyses were conducted for the broad and narrow selections of tests). Since modification indications capitalize on chance, we randomly divided these datasets into two halves; a developmental half and a cross-validation half. Our analysis was conducted in four steps; (1) examine the modification index output, (2) make the change suggested by the largest modification index, (3) obtain model fit in the developmental and cross-validation samples, and (4) repeat steps 1 to 3 if the ΔCFI in the cross-validation dataset is >0.01. Since making all of the modifications suggested by the modification indices will eventually approach a model with perfect fit, we set a stopping rule for ΔCFI so that only changes with meaningful increments in CFI were incorporated.

Making the modifications increased the fit of the higher-order model. For example, adding a correlated error between the Paragraph Comprehension and Word Knowledge/Vocabulary subtests in the 1979 ASVAB sample increased the cross-validated fit of the higher-order model from 0.904 to 0.922. However, when we made the corresponding modifications to the bifactor model (e.g., adding the correlated error between the two previously mentioned tests), the fit for the bifactor model also increased (e.g., from 0.961 to 0.980). After making all of the modifications, the bifactor model still had better cross-validated fit than the higher-order model using all of the fit statistics we studied for the three datasets. Thus, it appears that using modification indices to incorporate previously unmodeled complexities serves to increase the fit for both the higher-order and bifactor models. Indeed, there is overlap in the modification indices obtained using the higher-order and bifactor models. Using the developmental sample of the 1979 ASVAB data, we obtained the lists of modification indices with values of four or higher for the higher-order and bifactor models. The higher-order and bifactor models had 140 and 110 (respectively) modifications listed, of which 91 were in common. There were also sizable correlations between the modification index values for the two models (*r* = 0.43, *p* < 0.01; Spearman’s *ρ* = 0.34, *p* < 0.01) and the parameter changes values for the two models (*r* = 0.66, *p* < 0.01; Spearman’s *ρ* = 0.75, *p* < 0.01).

Turning to the pure factor cluster analyses, we ran the find one factor cluster (FOFC; Kummerfeld and Ramsey [54]; Kummerfeld, Ramsey, Yang, Spirtes, and Scheines [55]) algorithm in Tetrad V for each dataset (note that we ran FOFC separately for the broad and narrow selection of the ProjectTALENT tests). For 34 of the 50 datasets studied, FOFC did not yield any clusters of pure indicators. Although it is not a test of the bifactor model, a lack of clusters of pure indicators means that the higher-order model should be rejected (Yang et al. [53]). For 12 of the 50 analyses, FOFC only yielded one or two clusters, yet three are required to compare the higher-order and bifactor models. Yang et al suggest that some researchers may decide to reject the higher-order model in situations in which there are two few indicators, as is the case here for *g*. In the remaining four datasets, there were a sufficient number of clusters of pure indicators. However, creating CFAs for these clusters led to empirical identification issues in two datasets. This left two other datasets for which the bifactor and higher-order models could be compared using FOFC. In the Lucas and French [56] dataset, all of the fit statistics favored the higher-order model over the bifactor model. In the Thurstone and Thurstone [8] dataset, the *Δχ*^2^ test, NFI, and CFI indicated that the bifactor model had better fit, and TLI, RMSEA, and AIC indicated that the higher-order model had better fit. We did not assess each case’s contribution to chi-square to compare the higher-order and bifactor models as that analysis is not supported by the software we used. Taken as a whole, the results of these additional analyses provide more support for the bifactor model than for the higher-order model. However, future researchers should attempt to create and study test batteries with more factor purity.

### 3.2. Summary of Results

On average, across all 166 analyses, the bifactor model improved CFIs by 0.085 (median improvement 0.076), TLIs by 0.105 (median 0.083), NFIs by 0.099 (median 0.078), RMSEA by 0.027 (median 0.028), and AIC by 10,646 (median 1343). When a model had a factor with only two indicators, the bifactor model nearly always provided better fit than the higher-order model, regardless of how these factors were handled. As might be expected, the mixed and correlated error approaches to handling two-indicator factors performed better than models that dropped this factor or fixed the error terms to the reliabilities. The finding that the bifactor model provides better fit was exhibited using all three types of datasets; clinical intelligence test datasets, laboratory research datasets, and Industrial/Organizational employment-testing datasets. As an aside, some of the models had Heywood cases; negative variances commonly occurred for either fluid intelligence factors or its indicators. This is consistent with past research suggesting the possible (or near) equivalence of fluid intelligence and *g*. For example, Carroll ([9], p. 109) reports correlations at or near unity, and Reeve and Bonaccio [57] note that the variance for fluid intelligence often disappears after controlling for *g*. Similar arguments are made by Gustafsson [58,59].

## 4. Discussion

The bifactor model of mental abilities was originally hypothesized by Holzinger and Swineford [3,4]. After lying dormant for many years, researchers such as Gustafson [60] and Gignac [11,12,13,14] resurrected the study of the hypothesis and obtained preliminary support from several primary studies. Our synthesis of the existing primary studies and reanalysis of the published datasets has yielded support for the hypothesis that the bifactor model more accurately represents mental abilities than the higher-order model; *g* has its own loadings on subtest scores and is not a higher-order factor. To illustrate our findings, we found better support for the bifactor models shown in Figure 1b and Figure 2 than for the higher-order models shown in Figure 1a and the insets Figure 2.

Some might ask if our findings (i.e., that *g* is not a higher-order construct) are inconsistent with *g*-theory and the emphasis on *g* within the mental abilities literature. We do not believe this to be the case since, although *g* may not be a higher-order construct, it still accounts for a higher amount of variance in subtest scores and performance than other factors. This is evidenced by the exploratory factor analysis work showing that the first principal component or factor, which is viewed as one of the best operational measures of *g* (Jensen and Weng [7]; Ree and Earles [48]; Ree, Earles, and Teachout [49]) accounts for far more variance in scores in mental ability test batteries than any other component/factor. Additionally, the results using CFA reach a similar conclusion (Cucina and Howardson [61]).

Although conceptualizing *g* as a higher-order construct when conducting CFAs yields acceptable fit statistics, a bifactor conceptualization yields better fit both in terms of statistical significance (i.e., the Δ*χ*^2^ test) and effect size (e.g., ΔCFI). Even with the parsimony corrections for CFI, TLI, RMSEA, and AIC, the effect sizes, measured using the change in fit indices, were substantial enough to indicate that the bifactor model has better fit than the higher-order model. Ultimately, the choice between the bifactor and higher-order models depends on the end goal of the data analyst. In situations in which researchers only need a reasonable approximation of the factor structure of mental abilities, the higher-order model may be preferable. However, it is our interpretation and opinion that the bifactor model should be preferable to the higher-order model in most instances in which mental ability test scores are factor analyzed. In the following sections we discuss our interpretations, opinions, and comments on the bifactor (versus the higher-order) model, although we provide a caveat to this discussion with a recognition that other interpretations and positions on this topic are possible.

### 4.1. Loading Interpretation Considerations with the Higher-Order and Bifactor Models

The bifactor model also provides output that paints a more direct interpretation of factor loadings for *g* versus the broad factors. In the higher-order model, if *g* has a high loading on factor A and factor A has a high loading on subtest X’s scores, then it appears that factor A explains much of the variance in subtest X’s scores; however, it could be the case that factor A explains very little unique variance and instead is only allowing *g* to pass through it. This can present the illusion that the broad (and narrow, if modeled) factors account for substantial unique variance. Another way of conceptualizing this situation is to imagine an electrical power line emanating from a large power plant going to small power plant and then traveling to a city. The large power plant produces the bulk of the power, with the small power plant only contributing a minor amount. However, the amount of power in the lines between the two plants is large, as is the amount of power between the smaller power plant and the city. This creates the cursory illusion that both plants produce a lot of power, when in fact this is not the case.

Whenever the loading of a broad factor on *g* exceeds $\sqrt{0.50}$ in a higher-order model (and this is often the case), the unique portion of the broad factor explains less variance in subtest scores than *g*. Indeed, recent research using the WJI-III, KAIT, KABC, and DAS found that the broad factors account for only small amounts of variance in subtest scores, compared to *g*, which is consistent with Carroll’s (1993) Three Stratum Theory (Cucina and Howardson [61]). Consequently, researchers have noted that the more direct interpretation of factor loadings is a “chief virtue” (Reise [16], p. 674) of the bifactor model and that the provided output is “less ambiguous” (Canivez [62], p. 251; Chen, Hayes, Carver, Laurenceau, and Zhang [63], p. 223) than the higher-order model. Although the Schmid-Leiman [64] decomposition^2^ provides a method for separating these variances and Brown [27] shows how this can be easily implemented using a spreadsheet (or even a hand calculator), we note that this is too rarely done in practice.

### 4.2. Proportionality Considerations with the Higher-Order and Bifactor Models

**Figure 3:**
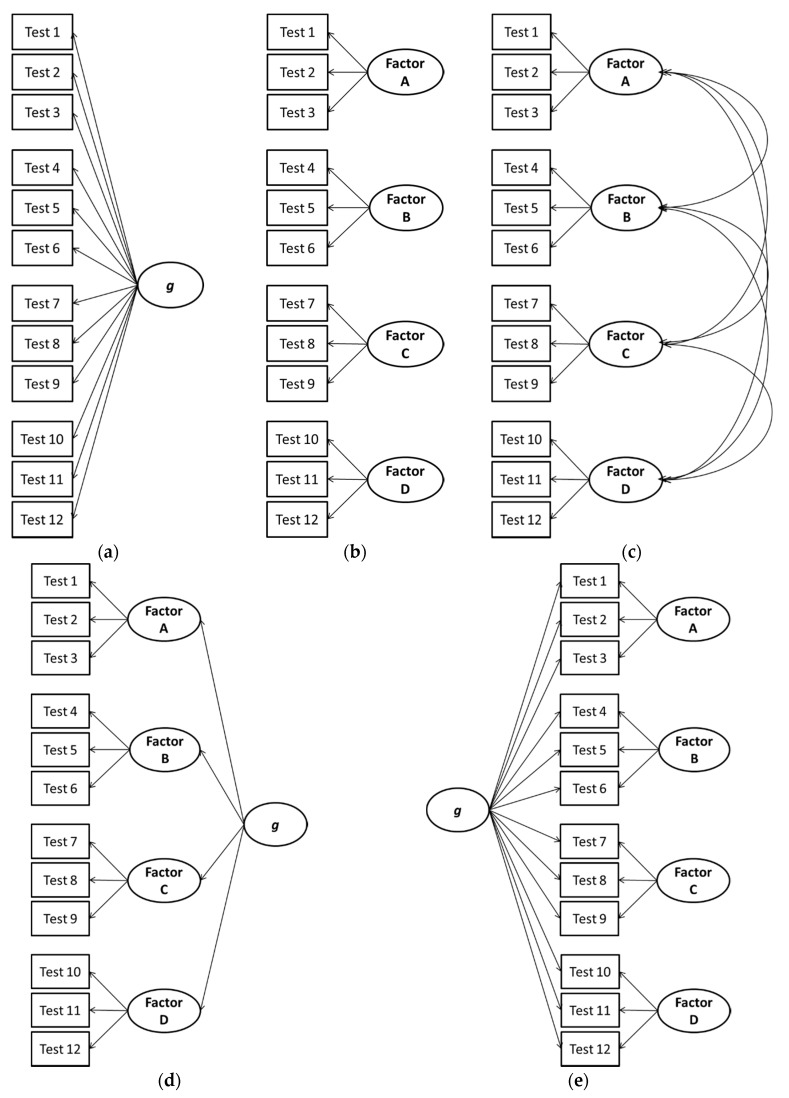
These figures shows the historical evolution of the theoretical models of the factor structure of mental abilities. The evolution begins with modeling of the general factor of mental abilities (*g*) in (**a**), followed by contentions that mental abilities were represented by multiple factors (**b**), and then a recognition that the multiple factors are correlated (**c**), followed by a realization that both *g* and broad factors existed (**d**). This is followed by the emergence of the bifactor model (**e**).

Gignac [19] notes that the Schmid-Leiman [64] decomposition makes the likely untenable assumption that the relationship between each subtest score and *g* is proportional within each factor due to a proportionality constraint associated with the higher-order model. The higher-order model introduces a proportionality constraint, meaning that the relationship between *g* and subtests 1, 2, and 3 in Figure 3d must be proportional to one another. This is because the only way that subtests 1, 2, and 3 can correlate with *g* is through the single *g*-loading between factor A and *g* (Yung, Thissen, and McLeod [52]). In contrast, the bifactor model includes no such constraint since subtest scores have independently estimated loadings on both *g* and the broad factors. Gignac [19] has provided evidence that violations of proportionality provide an explanation for why the bifactor model often has better fit statistics than the higher-order model. Essentially, when proportionality is violated, the bifactor model will provide better fit when parsimony corrections are not made (or when the degree of violation is large enough to offset parsimony corrections).

### 4.3. Parsimony Considerations with the Higher-Order and Bifactor Models

Murray and Johnson [18] have provided a good argument that the higher-order model is preferable due to its inherent parsimony. Indeed, parsimony is considered a desirable quality in a hypothesis. However, we suggest that an argument could be made for either the higher-order or bifactor model being more parsimonious. The higher-order model includes fewer paths and hence fewer parameters. Thus, from a fit statistics perspective, the higher-order model could be viewed as being more parsimonious. However, a case can also be made for the bifactor model being more parsimonious if one accepts a more general view of parsimony rather than strictly focusing on the number of parameters (as parsimony corrections for fit statistics do). We focus our discussion on the case for the parsimony of the bifactor model, as it has not been well described in the literature, to our knowledge. Consider the following equations representing the relationship between the subtests and factors. With the bifactor model, subtest scores are related to factors such as:

*Subtest_i_* = *a_i_* × *g* + *b_ij_* × *Factor_j_* + uniqueness

where *a_i_* is loading of subtest *i* on *g* and *b_ij_* is loading of subtest *i* on specific factor *j*

Under the higher-order model, two equations are required to depict the relationship between the subtests and factors (compared to one equation for the bifactor model):

*Subtest_i_* = *c_ij_* × *Factor_j_* + uniqueness

*Factor_j_* = *d_j_* × *g* + uniqueness

where *c_ij_* is loading of subtest *i* on specific factor *j* and *d_j_* is loading of specific factor *j* on *g*.

Thus the higher-order model could be viewed as complicating the relationship between subtest scores and *g* by introducing a mediating variable and constraining the loadings to be proportional. Although the bifactor model has more paths, these paths could be viewed as being less complex and more parsimonious than other types of changes to models (e.g., adding non-hypothesized paths, theory-inconsistent correlated errors, new *post hoc* factors). Given the primary role of *g* in predicting performance and the fact that it captures the most variance in scores in cognitive test batteries, we think it seems fitting to allow subtest scores to have direct loadings on *g*.

The higher-order model also complicates the relationship between scores on two individual subtests. Consider scores on the Vocabulary and Block Design subtests of the WAIS-III. These two subtests are positively correlated (*r* = 0.60; Colom et al. [65]); however, they load on different broad factors (verbal comprehension and perceptual organization, respectively, using Gignac’s [13] higher-order index model). The explanation of the correlation using a higher-order model is a bit roundabout. Vocabulary loads onto verbal comprehension, and Block Design loads onto perceptual organization. Both verbal comprehension and perceptual organization, in turn, load onto *g*. Thus Vocabulary correlates with Block Design because Vocabulary measures verbal comprehension, which correlates with *g*, which in turn correlates with perceptual organization, which in turn is measured by Block Design. A more parsimonious explanation is that both Vocabulary and Block Design load on *g*, and the positive correlations between the two are due to their loadings on *g*.

### 4.4. Is the Higher-Order Model a Remnant of an Antiquated Compromise?

We suggest that the higher-order model is most often presented in the literature due to the historical reasons that we depict in Figure 3. When Spearman [66,67] developed his theory of general intelligence, he showed that measures of different mental abilities tended to correlate with one another, which implies a general factor (see Figure 3a). This finding was met by challenges from psychologists who believed that intelligence consisted of multiple factors not a single factor, as shown in Figure 3b. For example, Thurstone [68] posited seven factors and rejected *g*. Eventually it became widely accepted that scores for multiple factors of intelligence correlated with one another, as shown in Figure 3c, which implies the higher-order general factor shown in Figure 3d. Thurstone [68] later accepted the existence and incorporation of *g* in his model at a higher-order level. The common analytical process was to correlate scores for the factors (either through correlating composite scores or through factor analysis) to identify a *g* factor. This process leaves the individual factors in place, allowing *g* to be added *post hoc* to the model later in the process.

We surmise that this approach serves as a *de facto* illusory compromise between proponents of the theory of a single mental ability and those of multiple intelligence factors. By allowing the extraction of multiple (albeit correlated) factors from a battery of mental ability subtests, the proponents of multiple intelligences were satisfied. By allowing these factors to load on a common general factor, the proponents of a general intelligence factor were satisfied. Unless a Schmid-Leiman [64] decomposition is performed, the loadings from *g* to the multiple factors and the loadings from the latter to the subtest scores are often quite high, providing cursory support for both positions. Given the results of our analyses, we believe that psychologists should being using the bifactor model shown in Figure 3e.

There is also a pragmatic reason for this compromise. A number of the mental ability test batteries were developed in the Twentieth Century using different theories of intelligence, some of which did not support the existence of *g*. By adding *g* to these test batteries on the back end, a multiple-intelligence conceptualization could remain intact while also satisfying proponents of *g*. As is the case now, for operational reasons, it is difficult to change test batteries that are currently in use, as this presents score comparability issues and logistical issues. Additionally, the consumers of mental ability subtest scores are often not trained psychologists; instead they tend to be patients, parents, courts, employers, and school officials who are often more open to theories of multiple intelligences than are trained psychologists.

### 4.5. Limitations and Future Research

We were unable to include tests from several large-scale testing programs (e.g., the SAT) since these tests include too few subtest scores to form factors. Future researchers could study these tests if these were administered as part of a joint CFA study. We were also unable to locate meta-analytic procedures for combining fit indices from different datasets to summarize analyses. When meta-analytic CFAs/SEMs are conducted, a meta-analytic correlation matrix is produced, which is then subjected to CFA/SEM. In our study, the datasets included different test batteries and factors, making it impossible to create a complete meta-analytic matrix. We were unable to find a published method for combining fit statistics from different datasets via meta-analysis. Future methodological researchers could explore the possibility of combining fit indices from multiple studies using meta-analysis. Additionally, to date, no test battery has been developed that measures all of Carroll’s [9] abilities. Such a battery would allow for a better test of the bifactor model; however, cost and examinee fatigue could be issues. Our finding that the bifactor model provides better model fit is largely limited to the test batteries, subtests, and broad factors that were included in the datasets we studied. It is possible that other broad factors are best modeled by higher-order models. On a related note, we largely used existing factor analytic models (e.g., the models suggested by previous researchers who used the datasets or a conceptual linkage of the subtests to Carroll’s [9] work). Therefore, the conceptualization of the broad factors differed between the datasets we studied. Furthermore, future research could also examine the possibility that a bifactor model can be applied at the broad factor level; that is, is the relationship between broad factors and subtests mediated by narrow factors? Finally, an examination of the usefulness of the bifactor model in applied settings would be interesting (i.e., does a bifactor model give paths from *g* to a criterion such as job performance or clinical outcomes that differ noticeably than those from a higher-order model?). It may be the case that, for applied purposes, the bifactor model does not have any statistical advantages (e.g., improved prediction of outcomes) over the higher-order model, despite having better model fit.

## Figures and Tables

**Table 1:** Summary of Fit Statistics from Past Bifactor Research.

Citation	Battery/Notes	Higher-Order								Comparison			Bifactor							
		CFI	TLI	NFI	RMSEA	AIC	*χ*^2^	*df*	*p*	*Δχ*^2^	*df*	*p*	CFI	TLI	NFI	RMSEA	AIC	*χ*^2^	*df*	*p*
Beaujean et al. [21]	WISC-IV	0.981	0.976	0.960	0.039	226.74	150.74	82	<0.001	23.15	9	0.006	**0.985**	**0.978**	**0.966**	**0.037**	**221.60**	127.60	73	<0.001
Benson et al. [22]	WISC-IV	0.956	0.950	0.925	0.042	1108.74	934.74	409	<0.001	122.14	24	<0.001	**0.964**	**0.957**	**0.934**	**0.039**	**1034.60**	812.60	385	<0.001
Gignac & Watkins [23]	WAIS-IV ^a^																			
	Age 16-19	0.945	0.933	0.918	0.068	314.75	246.75	86	<0.001	99.47	11	<0.001	**0.975**	**0.965**	**0.951**	**0.049**	**237.28**	147.28	75	<0.001
	Age 20-34	0.959	0.950	0.944	0.064	366.51	298.51	86	<0.001	101.3	11	<0.001	**0.977**	**0.967**	**0.963**	**0.052**	**287.21**	197.21	75	<0.001
	Age 35-54	0.943	0.930	0.920	0.075	347.28	279.28	86	<0.001	118.85	11	<0.001	**0.975**	**0.965**	**0.954**	**0.053**	**250.43**	16.43	75	<0.001
	Age 55-69	0.948	0.937	0.927	0.074	341.93	273.93	86	<0.001	78.98	11	<0.001	**0.967**	**0.954**	**0.948**	**0.063**	**284.95**	194.95	75	<0.001
Gignac [11]	WAIS-R ^b^	0.970	0.959	0.967	0.068	443.97	391.97	40	<0.001	229.69	7	<0.001	**0.989**	**0.982**	**0.986**	**0.046**	**228.28**	162.28	33	<0.001
Gignac [13]	WAIS-III ^c^	0.968	0.959	0.965	0.064	723.38	663.38	61	<0.001	215.13	10	<0.001	**0.979**	**0.968**	**0.976**	**0.056**	**528.25**	448.25	51	<0.001
Gignac [12]	MAB ^d^	0.955	0.941	0.953	0.077	664.9	622.9	34	<0.001	237.56	9	<0.001	**0.973**	**0.951**	**0.971**	**0.069**	**445.34**	385.34	25	<0.001
Gignac [14]	Colom S1 ^e,f^	0.893	**0.859**	0.815	**0.073**	**157.8**	101.8	50	<0.001	14.65	8	0.066	**0.907**	0.854	**.842**	0.074	159.15	87.15	42	<0.001
	Colom S2 ^e^	0.913	0.893	0.783	0.049	196.48	126.48	85	0.002	28.64	10	0.001	**0.952**	**0.933**	**0.832**	**0.039**	**187.84**	97.84	75	.039
	Colom S3 ^e^	0.878	0.850	0.767	0.064	221.1	151.1	85	<0.001	21.84	10	0.016	**0.900**	**0.860**	**0.800**	**0.061**	**219.26**	129.26	75	<0.001
	G1984 ^g^	0.958	0.951	0.943	0.051	673.15	575.15	161	<0.001	192.78	17	<0.001	**0.976**	**0.968**	**0.962**	**0.041**	**514.37**	382.37	144	<0.001
	HS1939/G2001 ^h^	0.901	0.890	0.827	0.060	617.27	511.27	247	<0.001	76.98	19	<0.001	**0.923**	**0.907**	**0.853**	**0.055**	**578.29**	434.29	228	<0.001
Golay & Lecerf [24]	French WAIS-III ^i^	0.965	0.956	0.957	0.059	359.5	301.5	62	<0.001	178.5	9	<0.001	**0.990**	**0.985**	**0.983**	**0.035**	**199**	123	53	<0.001
Niileksela et al. [25]	WAIS-IV	0.964	**0.967**	0.942	0.067	193.62	179.62	71	<0.001	10.76	5	0.056	**0.966**	0.966	**0.945**	**0.062**	**192.86**	168.86	66	<0.001
Von Stumm et al. [26]	Lab Study ^j^	0.934	0.905	0.897	0.079	103.19	63.19	25	<0.001	29.2	4	<0.001	**0.977**	**0.962**	**0.944**	**0.050**	**81.99**	33.99	21	0.036

Notes: Tests for which the bifactor model had significant incremental fit are in blue, and those for which the higher-order model had better fit are in red. For each fit index, we highlight the better fitting model using bold font. CFI: Comparative Fit Index; TLI: Tucker-Lewis Index; NFI: Normed Fit Index; RMSEA: Root Mean Square Error of Approximation; AIC: Akaike Information Criterion. Values are reprinted from the original studies. When the original studies did not provide a fit statistic, the formulas provided in Brown [27] and Mueller [28] were used. For example, Beaujean et al. [21] did not provide TLI and NFI in their original paper; however, CFI, χ^2^, and *df* were provided for the hypothesized model. We estimated the values of TLI and NFI using a three-step process. First, we computed *df_null_* using the number of observed variables (i.e., subtest scores). Next, we located the formula for CFI given in Mueller [28] and solved this formula for the value of χ_null_^2^. We assumed that χ_null_^2^ – *df_null_* > χ_hypothesized_^2^ – *df_hypothesized_* > 0 when solving. Next we computed TLI and NFI using the formulas provided by Mueller [28]. We computed all AICs using the formula used by AMOS and LISREL (i.e., *χ*^2^+2 (number of free parameters)), which Brown [27] describes on p. 175. ^a^ This is the fourth revision of the Wechsler Adult Intelligence Scale (WAIS; Wechsler [29]), a popular clinical intelligence test. ^b^ This is the second revision of the WAIS (Wechsler [30]). ^c^ This is the third revision of the WAIS (Wechsler [31]). ^d^ The Multidimensional Aptitude Battery (MAB; Jackson [32,33]) is a group-administered mental abilities test. The MAB is a general-purpose test battery that is used in employment, research, and clinical settings. ^e^ Gignac [14] conducted these analyses on correlation matrices from three samples in studies by Colom et al. [34]. ^f^ Gignac [14] reported a *df* of 50 for both the null and higher-order models; we assumed a value of 66 for the *df* of the null model based on the number of tests/measured variables (note *df* = *k*(*k* – 1)/2) and the fact that the higher-order model requires the estimation of 16 more parameters than the null model does. ^g^ Gignac [14] also analyzed a correlation matrix from Gustafson [35]. ^h^ Gignac [14] also analyzed the correlation matrix from Holzinger and Swineford’s [4] study; this matrix is published in Gustafson [36]. ^i^ This is the French-language version of the fourth revision of the WAIS. ^j^ Von Stumm et al. [26] administered nine tests: the Ravens Advanced Progressive Matrices (Raven [37]), Yela’s [38] rotation of solid figures test, three of Thurstone’s [39] Primary Mental Abilities tests, and four tests from the Differential Aptitude Tests [40,41].

**Table 2:** χ^2^ Tests Comparing Observed Counts of Fit Statistic Results Across all 166 Analyses to those Expected from Morgan et al.’s [43] Monte Carlo Simulation.

		True Model = Bifactor; Fitted Model = Bifactor *^a^*			True Model = Higher-Order; Fitted Model = Bifactor *^b^*		
Factor Structure *^c^*	*n ^d^*	CFI	TLI	RMSEA	CFI	TLI	RMSEA
**Expected Results *^e^***							
3:1 & 2:1	200	156	151	148	120	111	111
3:1 & 2:1	800	166	166	166	133	118	108
3:1	200	151	143	141	120	110	106
3:1	800	166	166	166	138	121	110
**Observed Results *^f^***							
3:1 & 2:1	200	165	156	156	165	156	156
3:1 & 2:1	800	165	156	156	165	156	156
3:1	200	165	156	156	165	156	156
3:1	800	165	156	156	165	156	156
***χ*^2^*^g^***							
3:1 & 2:1	200	0.5	0.2	0.4	16.9	18.2	18.2
3:1 & 2:1	800	0.0	0.6	0.6	7.7	12.2	21.3
3:1	200	1.3	1.2	1.6	16.9	19.2	23.6
3:1	800	0.0	0.6	0.6	5.3	10.1	19.2
***p***							
3:1 & 2:1	200	0.471	0.684	0.511	<0.001	<0.001	<0.001
3:1 & 2:1	800	0.938	0.438	0.438	0.006	<0.001	<0.001
3:1	200	0.255	0.277	0.207	<0.001	<0.001	<0.001
3:1	800	0.938	0.438	0.438	0.022	0.001	<0.001

*^a^* This set of columns pertains to Morgan et al.’s [43] simulations that used a bifactor model to analyze data that were generated to be truly bifactor. *^b^* This set of columns pertains to Morgan et al.’s [43] simulations that used a bifactor model to analyze data that were generated to be truly higher-order. *^c^* Indicators per factor in Morgan et al.’s [43] study included two conditions; (1) a condition in which two factors had three indicators each and two other factors had two indicators each and (2) a condition in which four factors had three indicators each. We included the results for both conditions in our analyses. *^d^* Morgan et al.’s [43] study included two sample sizes (*n* = 200 and 800); we included the results for both in our analyses. *^e^* The expected results were computed by multiplying the percentages reported in Morgan et al.’s [43] study by the 166 comparisons used here. *^f^* The observed results were the number of times each fit statistic identified the bifactor model as having superior fit. Instances in which the fit statistics were equal were treated as support for the higher-order model. *^g^ df* = 1.

## Footnotes

1 We prefer the term bifactor since alternative meanings of the words “hierarchical” (cf. the higher-order model shown in Figure 1 and Figure 2 with most hierarchical organizational trees/charts) and “nested” (which often implies that cases are wholly nested in a larger class similar to the narrow factors being nested in the higher-order factors).

2 To briefly explain, the Schmid-Leiman [64] decomposition can be applied to a higher-order model, whereby a *g*-loading can be computed for each test by multiplying the loading of the test on the broad factor (i.e., λ*_TiFi_*, where *T_i_* is the test *i* and *F_j_* are factor *j*) by the loading of the broad factor on the higher-order *g* factor (i.e., λ*_Fig_*), resulting in the following formula: *g*-loading = λ*_Fig_* × λ*_TiFi_*. As we discussed earlier, the *g*-loadings for tests are constrained in higher-order models, since they can only take on values that are proportional to the loading of the broad factor on the higher-order *g* factor (λ*_Fig_*). Thus, the higher-order model can be viewed as a version of the bifactor model in which the *g*-loadings are fixed, not to a specific value but to proportional values.

## Supplementary Materials

The following are available online at http://www.mdpi.com/2079-3200/5/3/27/s1, Table S1: Description of tests and linkages of subtests to factors, Table S2: Description of datasets, Table S3: Fit statistics for best fitting models for each dataset.
