# Supplementary material for: The Bifactor Model Fits Better Than the Higher-Order Model in More Than 90% of Comparisons for Mental Abilities Test Batteries
Source: J Intell. 2017 Jul 11;5(3):27. doi: 10.3390/jintelligence5030027 (PMC6526460; doi:10.3390/jintelligence5030027)
Supplement: Supplementary file 1 [file jintelligence-05-00027-s001.pdf]

Journal of *Intelligence*

Supplementary Materials

The Bifactor Model Fits Better Than the Higher-Order Model in More Than 90% of Comparisons for Mental Abilities Test Batteries

Jeffrey Cucina and Kevin Byle

Table S1. Description of tests and linkages of subtests to factors.

Abbreviation	Test	Description	Subtest-Factor Linkage[a]
AFOQT	Air Force Officer Qualifying Test (U.S. Air Force, n.d.)	This test battery is used by the U.S. Air Force to select applicants for commissioned officer, pilot, and navigator positions. The AFOQT is a group-administered multiple-choice test that is administered in proctored settings.	Carretta and Ree's (1995, CR95) factor structure was used. Note that primary loadings are shown in **bold** font and secondary loadings are shown in normal font.<br><br>Math (M): **Arithmetic Reasoning (AR), Data Interpretation (DI), Math Knowledge (MK),** Scale Reading (SR)<br>Perceptual Speed (PS): **SR, Block Counting (BC), Table Reading (TR),** DI<br>Spatial (S): **Electrical Maze (EM), Rotated Blocks (RB), Hidden Figures (HF),** Mechanical Comprehension (MC), BC<br>Technical Knowledge (TK): **MC, Instrument Comp. (IC), Aviation Information (AI), General Science (GS)**<br>Verbal (V): **Verbal Analogies (VA), Reading Comprehension (RC), Word Knowledge (WK),** GS
ASVAB	Armed Services Vocational Aptitude Battery (U.S. Defense Manpower Data Center, 2010)	The ASVAB is one of the largest group-administered multiple-aptitude test battery programs in the world and is used by the U.S. military for selecting applicants into the U.S. Armed Forces and assigning them to military positions. This test uses a multiple-choice item format and is administered in proctored settings using either paper-and-pencil testing or computer adaptive testing. By Federal law, a minimum score on the Armed Forces Qualifying Test (which is a composite of a subset of the	Ree and Carretta's (1994; RC94) factor structure was used.<br><br>Technical Knowledge Factor (TKF): General Science (GS), Auto Information (AI), Shop Information (SI), Mechanical Comprehension (MC), Electronics Information (EI), Assembling Objects (AO).<br>Verbal/Math Factor (VMF): Arithmetic Reasoning (AR), Word Knowledge/Vocabulary (WK), Paragraph Comprehension (PC), Mathematics Knowledge (MK)<br>Speed Factor (SF): Numerical Operations (NO), Coding Speed (CS),

Abbreviation	Test	Description	Subtest-Factor Linkage[a]
		ASVAB tests) is required for entrance into the U.S. Military. The ASVAB is also used by high school counselors and educators to assist students as part of the no-cost Career Exploration Program.	
BAS3	British Ability Scales (Elliott, 2011)	The BAS3 (previously named the British Intelligence Scale; the U.S. version is the DAS) is an off-the-shelf mental abilities test battery. The BAS3 is primarily used to assess children, particularly those with learning disabilities.	The BAS3 tests were rationally linked to McGrew's (2005) and Carroll's (1993) factors by the authors. Since the Gs factor only had one indicator, it was omitted and its test loaded only on *g*.<br><br>Crystallized Intelligence (Gc): Word Definitions (WD), Verbal Similarities (VS), Spelling (S), Word Reading (WR)<br>Broad Visual Perception (Gv): Matrices (M), Recall of Designs (RD), Pattern Construction: Standard (PCS), Pattern Construction: Alternate (PCA), Recognition of Pictures (ROP)<br>Quantitative Knowledge (Gq): Quantitative Reasoning (QR), Number Skills (NS)<br>Short-Term Memory (Gsm): Recall of Digits: Forward (RDF), Recall of Digits: Backward (RDB), Recall of Objects: Immediate Verbal (ROIV), Recall of Objects: Immediate Spatial (ROIS), Recall of Objects: Delayed Verbal (RODV), Recall of Objects: Delayed Spatial (RODS)<br>Broad Cognitive Speediness (2S): Speed of Information Processing (SIP),
COGAT	Cognitive Abilities Test (Lohman & Hagen, 2002)	The CogAt is a mental abilities test battery used to assess K-12 abilities in reasoning and problem solving.	The publisher's factor model was used.<br><br>Verbal: Verbal Classification (VC), Sentence Completion (SC), Verbal Analogies (VA)<br>Quantitative: Quantitative Relations (QR), Number Series (NS), Equation Building (EB)<br>Nonverbal: Figure Classification (FC), Figure Analogies (FA), Figure Analysis (FAS)
DAS	Differential Ability Scales (Elliott, 1990)	The DAS is an off-the-shelf cognitive ability testing battery that consists of 22 subtests. The DAS is used to assess cognitive abilities that are related to important aspects of learning during development (ages 2-18).	The factor-linkages from the DAS manual were used.<br><br>Crystallized Intelligence (Gc): Word Definitions (Wdef), Spelling (Spel), Word Reading (WR)<br>Fluid Intelligence (Gf): Similarities (Sim), Matrices (Mat), Sequential and Quantitative Reasoning (SQR), Basic Number (BNS)

Abbreviation	Test	Description	Subtest-Factor Linkage[a]
			General Speediness (Gs): Speed of Information Processing (SIP)<br>General Short-Term Memory (Gsm): Recall of Digits (Rdig)<br>General Visualization (Gv): Recall of Designs (Rdes), Pattern Construction (PC), Pattern Construction Alternative (PCA), Recall of Objects-Immediate (Robi), Recall of Objects-Delayed (Robd), Recognition of Pictures (Rpic)<br>Note that Robi and Robd also link to General Long-Term Memory (Glr), which was modeled using a correlated error term.
DAT	Differential Aptitude Tests (Psychological Corporation, 1991; Bennet, Seashore, & Wesman, 1990).	The DAT is an off-the-shelf mental abilities test battery consisting of eight tests. The DAT is used for career counseling, personnel selection, and admissions to occupational training programs (e.g., trade schools).	Two factor-linkages are described in the results. The first came from Table 7.4 (p. 30) of the DAT manual and is as follows:<br><br>General Reasoning (R): Verbal Reasoning (VR), Numerical Ability (NA), Abstract Reasoning (ARDAT), Space Relations (SR)<br>Mechanical Operations and Principles (M): Mechanical Reasoning (MR)<br>Verbal Achievement (V): Spelling (S), Language Usage (LU)<br>Clerical Speed (C): Clerical Speed and Accuracy (CSA)<br><br>The second factor-linkage assigned the DAT tests to the ASVAB factors from Ree and Earles (1990):<br><br>TKF: MR, SR<br>VMF: VR, NA, ARDAT, SP, LU<br>SF: CSA
Detterman (1985)	Research-based test battery, related to ASVAB	This is an unpublished study of learning rates consisting of scores for multiple tests.	The following factor structure, developed by Carroll (1993), was used:<br><br>Factor 2: FAC, Lrpcal Prop correct All Trials, Rcncor Prop Correct Responses, Rlsmat Jack's Trials Corr Early, Spprco Prop Resp Corr All, Tdmdth Median Threshold Units, Tdnurd # Correct Diff Resp's, Ttmdth Median Threshold Units, Ttnurd # Correct Diff. Resp's, Ttnurs # Correct Same Resp's<br>Factor 3: Lrpcor Jack's Corr'd P Correct, Lrrtall Mean RT All Trials, Rcmndt Mean Dt All Trials,<br>Rtdt Mean Decis'n Time All Trls, Rtmtin Intercept MT All Trials<br>Factor 4: Rcsdmt Sd Mt All Trials, Sdmndt Mean Dt All Trials

Abbreviation	Test	Description	Subtest-Factor Linkage[a]
			Factor 5: Sdm Cmt Mean Mt Correct Trials, Stscpm Slope Mt Corr Pres Trials, Tdmnmt Mean Mt All Trials, Ttmnmt Mean Mt All Trials<br>Factor 7: Spmnrtp1 Mean Rt Position 1, Spmnst mean Study Time All Pos, Spmnstp7 Mean Study Time Pos 7<br>Factor 8: Sticad Interc Dt Corr Abs Trls, Stmntt Mean Trial Time All Trials<br>Factor 9: Tdsddt Sd Dt All Trials, Ttmndt Mean Dt all Trials<br>Factor 10: Prpcp6 Prop Correct Posn 6, Prs Dtt Sd Trial Time All Trials, Prsdrt Sd Rt All Trials
EAS	Employee Aptitude Survey (Ruch, Stang, McKillip, & Dye, 1994)	The EAS is an off-the-shelf test battery used for personnel selection. It includes 10 paper-and-pencil tests and the manual reports meta-analytic criterion-related validity evidence as well as construct validity evidence using other mental abilities tests.	The EAS tests were rationally linked to Carroll's (1993) factors by the authors.<br><br>Crystallized Intelligence (Gc): Verbal Comprehension (EAS1)<br>Broad Cognitive Speediness (Gs): Numerical Ability (EAS2), Visual Speed and Accuracy (EAS4), Manual Speed and Accuracy (EAS9)<br>Broad Visual Perception (Gv): Visual Pursuit (EAS3), Space Visualization (EAS5)<br>Fluid Intelligence (Gf): Numerical Reasoning (EAS6), Verbal Reasoning (EAS7), Symbolic Reasoning (EAS10)<br>Broad Retrieval Ability (Gr): Word Fluency (EAS8)
GATB	General Aptitude Test Battery (U.S. Department of Labor, 1958)	The GATB was a group-administered paper-and-pencil multiple abilities test that was developed by the U.S. Department of Labor. The purpose of the GATB was to provide state and local employment agencies with a test that could be used to refer applicants to local employers.<br><br>Perhaps no other test has had such a profound impact on the current state of personnel selection than the GATB. This test served as the initial basis for Schmidt and Hunter's (2002) seminal validity generalization work. Due to concerns about adverse impact, the National Academy of Sciences was asked to study the validity and fairness of the GATB, producing a somewhat controversial report which, nonetheless, established the fairness of cognitive ability tests for different groups	Three datasets used three different versions of the GATB. For the GATB B1001 and B1002 datasets, the tests were rationally linked to Carroll's (1993) and McGrew's (2009) factors.<br><br>GATB from B1001 Dataset<br>Broad Cognitive Speediness (Gs): Tool Matching, Name Comparison, Computation, Form Matching.<br>Broad Visual Perception (Gv): Two-Dimensional Space, Three-Dimensional Space<br>Fluid Intelligence (Gf): Arithmetic Reasoning<br>Crystallized Intelligence (Gc): Vocabulary<br>Motor: H Markings, Speed, Mark Making, Place, Turn, Assemble, Disassemble.<br><br>GATB from B1002 Dataset

Abbreviation	Test	Description	Subtest-Factor Linkage[a]
		(Hartigan & Wigdor, 1989). The GATB use of within-group norming influenced the outlawing the practice in the 1991 Civil Rights Act.	Crystallized Intelligence (Gc): Verbal Aptitude<br>Fluid Intelligence (Gf): Numerical Aptitude<br>Broad Cognitive Speediness (Gs): Numerical Aptitude, Form Perception, Clerical Perception<br>Motor: Motor Coordination, Finger Dexterity, Manual Dexterity<br><br>The third dataset was from a joint administration of the ASVAB and the GATB. Peterson (1993, p. 17) reports factor analysis results and linkages (based on work by Wise and McDaniel, 1991) for a similar study.<br><br><u>ASVAB-GATB Kettner (1997) Dataset using Peterson linkages</u><br>Note: primary linkages are in **bold** font and secondary linkages are in regular font.<br>Verbal: **ASVAB-General Information (GI), Word Knowledge (WK), General Science (GS), General Biological Science (GB); GATB-Vocabulary (VO)**<br>Speed: **ASVAB-Numerical Operation (NO), Attention to Detail (AD);** GATB – Mark Making (MM)<br>Quantitative: ASVAB-Arithmetic Reasoning (AR), Math Knowledge (MK); GATB – Computation (CO), Arithmetic Reasoning (AR)<br>Technical: **Space Perception (SP), Electronic Information (EI), Mechanical Comprehension (MC), Shop Information (SI), Automotive Information (AI),** GS, GB; **GATB – Dimensional Space (SI)**<br>Perceptual: ASVAB – SP; **GATB – Name Comparison (NC), Tool Matching (TM), Form Matching (FM), Mark Making (MM),** SI
George Washington Mental Alertness and Social Intelligence Test	Thorndike (1936)	In a research study, Thorndike (1936) examined the factor structure of the George Washington Mental Alertness and Social Intelligence Test	The following factor structure, developed by Carroll (1993), was used:<br><br>F2: Comprehension, Memory for Names and Faces, Sense of Humor<br>F3: Vocabulary, Learning Ability, Judgment in Social Situations, Recognition of Mental State, Observation of Human Behavior<br>F4: General Information, Arithmetical Reasoning
Guilford &	Guilford & Lacey	Research-based Aviation Classification Test	The following factor structure, developed by Carroll (1993), was used:

Abbreviation	Test	Description	Subtest-Factor Linkage[a]
Lacey	(1947)		F2: Judgment, Mechanical Judgment Information in Judgment, Mechanical Comprehension, Mechanical Movements<br>F4: Vocabulary, Commonsense/Pure Judgment, Reasoning in Reading, Syllogisms<br>F3: Logical Reasoning Judgment, Deductive Reasoning, Arithmetical Reasoning<br>F5: Figure Analogies, Pattern Reasoning
Gustafsson	Gustafsson (1984)	Research-based test battery	The factor linkages were obtained from the original article.<br><br>Visualization (Vz): Metal Folding – Odd Items, Metal Folding – Even Items<br>Spatial Orientation (S): Card Rotation – Part 1, Card Rotation – Part 2,<br>Flexibility of Closure (Cf): Group Embedded Figures, Hidden Patterns, Copying<br>Speed of Closure (Cs): Disguised Words, Disguised Pictures<br>Cognition of Figural Relations (CFR): Raven – Odd Items, Raven – Even Items<br>Induction (I): Number Series, Letter Grouping<br>Memory Span (Ms): Auditory Number Span, Auditory Letter Span<br>Verbal Comprehension (V): Opposites – Odd Items, Opposites – Even Items<br>Achievement: Swedish Achievement, Mathematics Achievement, English Achievement.
IST	Intelligence Structure Test (Liepmann, Beauducel, Brocke, & Horn, 2001; Liepmann, Beauducel, Brocke, & Amthauer, 2007)	This is an intelligence test battery that can be administered either individually or in a group setting. Based on Beauducel, Brocke and Liepmann's (2001) structural model for intelligence, this test is primarily used in Europe.	The factor-linkages were obtained from the IST-R manual.<br><br>Verbal (V): Sentence Completion (SC), Verbal Analogies (VA), Verbal Similarities (VS)<br>Numerical (N): Calculations (CA), Number Series (NS), Numerical Signs (SI)<br>Figural: Figure Selection (FS), Cubes (CU), Matrices (MA)
Johnson, Nijenhuis, and Bouchard	Johnson, Nijenhuis, & Bouchard (2008).	Battery of Multiple Tests	Johnson et al.'s (2008) factor structure was used; however, a technical knowledge factor was added and linked to 2 tests. Tests were used from the Test Battery of the Royal Dutch Navy, TIB Battery, Cattell Culture Fair Test, GATB, and the Groninger Intelligentie Test.

Abbreviation	Test	Description	Subtest-Factor Linkage[a]
			Building: Test 28-Three-dimensional Space, Test-36 Finger Dexterity Board: Assemble<br>Closure: Test 38-Word List, Test 40-Gestalt Completion, Test 42-Mutilated Words<br>Dexterity: Test 33-Mark Making, Test 34-Pegboard Manual Dexterity-Place, Test 35-Pegboard Manual Dexterity-Turn, Test 36-Finger Dexterity Board-Assemble, Test 37-Finger Dexterity Board-Disassemble<br>Fluency: Test 43-Naming Animals, Test 44-Naming Professions<br>General: Test 22-Test 1-Series, Test 23-Test 2-Classification, Test 24-Test 3-Matrices, Test 25-Test 4-Conditions (topology)<br>Identification: Test 10-Parts, Test 12-Judgment, Test 14-Blocks, Test 13-Dimension<br>Mechanical Ability: Test 1-Mechanical Comprehension, Test 6-Form Perception<br>Memory: Test 11-Precision, Test 18-Memory,<br>Test 9-Tools, Test 16-Perception<br>Organizing: Test 39-Figures, Test 41-Sorting<br>Perceptual Speed: Test 5-Administrative Ability, Test 7-Administrative Speed, Test 8-Four Letter Words<br>Problem Solving: Test 2-Verbal, Test 3-Computation Part 1, Test 4-Computation Part 2<br>Reasoning: Test 13-Dimension, Test 15-Numbers, Test 17-Fluency 1 and 2, Test 16-Perception<br>Spatial: Test 30-Tool Matching , Test 32-Form Matching<br>Speed: Test 19-Maze, Test 20-Checks, Test 21-Dots<br>Verbal: Test 26-Name Comprehension, Test 27-Computation, Test 29-Vocabulary, Test 31-Arithmetic Reasoning<br>Technical Knowledge: Test 45-Dial Reading, Test 46-Table Reading Part 1
KABC	Kaufman Assessment Battery for Children – Second Edition (Kaufman & Kaufman,	The KABC is an off-the-shelf cognitive ability testing battery that consists of 18 subtests. The KABC is primarily used as a diagnostic test for cognitive development for individuals ages 3-18.	The factor-linkages were obtained from the KABC Manual.<br><br>General Short-Term Memory (Gsm): Number Recall (NR), Word Order (WO), Hand Move (HM)

Abbreviation	Test	Description	Subtest-Factor Linkage[a]
	2004)		General Visualization (Gv): Rover (RO), Block Counting (BC), Triangles (TR), Gestalt Closure (GEC)<br>General Long-Term Memory (Glr): Rebus (RE), Atlantis Delayed (AD), Rebus Delayed (RD)<br>Fluid Intelligence (Gf): Pattern Reasoning (PR), Story Completion (SC)<br>Crystallized Intelligence (Gc): Riddles (RI), Verbal Knowledge (VK), Expressive Vocabulary (EV)
KAIT	*Kaufman Adolescent & Adult Intelligence Test.* (Kaufman & Kaufman, 1993)	The KAIT is a general intelligence test that is used to assess fluid and crystallized intelligence in adolescents and adults (ages 11 to 85).	The factor-linkages were obtained from the KAIT Manual.<br><br>Crystallized Intelligence (Gc): Definitions (Def), Auditory Comprehension (AudCom), Double Meanings (DblMean), Famous Faces (Faces).<br>General Long-Term Memory (Glr): Rebus Learning (RebLrn), Rebus Delayed Recall (RebDR), Auditory Delayed Recall (AudDR)<br>Fluid Intelligence (Gf): Logical Steps (LogStep), Mystery Codes (MystCod)<br>General Visualization (Gv): Memory for Block Designs (Block)
LAMP	Learning Abilities Measurement Program (Sawin, Earles, & Goff, 2001)	The LAMP was a multiple ability test battery that was developed by the U.S. Air Force. Created using a cognitive psychology framework, the LAMP includes tests measuring working memory, skill learning, induction, and fact learning. The tests included in our current study are a subset of the 59 tests that were developed as part of the LAMP. Although Sawin, Earles, and Goff (2001) state that the LAMP was disbanded due to budget cuts in the late 1990s, the LAMP did lead to a number of publications (Kyllonen, 1993, 1994; Kyllonen & Allusi, 1987; Kyllonen & Christal, 1989, 1990; Roznowski et al., 2000).	In the joint-factor-analyses with the ASVAB, the ASVAB factor-linkages described above were used and the below LAMP factor-linkages were added to the model.<br><br>Working Memory (WM): WM-Quantitative (WMQ), WM-Spatial (WMS), WM-Verbal (WMV)<br>Skill Learning (SL): SL-Quantitative (SLQ), SL-Spatial (SLS), SL-Verbal (SLV)<br>Induction (IN): IN-Quantitative (INQ), IN-Spatial (INS), IN-Verbal (INV)<br>Fact Learning (FL): FL-Quantitative (FLQ); FL-Spatial (FLS), FL-Verbal (FLV)
Lucas & French (1953)	Lucas & French (1953)	Research-based Naval Test Battery	Lucas and French's (1953) factor structure was used and a second factor model was created by rationally linking the tests to McGrew's (2005) and Carroll's (1993) factors.<br><br><u>Lucas and French (1953) factor structure:</u>

Abbreviation	Test	Description	Subtest-Factor Linkage[a]
			Factor 1: Test 10-Addition, Test 11-Division, Test 12-Subtraction and Multiplication<br>Factor 2: Test 5-Vocabulary, Test 6-Verbal Reasoning, Test 25-Time and Distance Estimations<br>Factor 3: Test 15-Similar Rotations, Test 16-Paper Folding, Test 17-Surface Development, Test 7-Block Assembly, Test 8-Block Recognition, Test 9-Similar Figures<br>Factor 4: Test 13-Cancellation, Test 14-Number Checking<br>Factor 5: Test 2-Figure Analogies, Test 23-Spatial Rotation, Test 24-Artificial Language<br>Factor 6: Test 1-Mathematics, Test 18-Relative Movement<br>Factor 7: Test 19-Operations, Test 20-Directional Plotting, Test 21-Plotting, Test 28-Skywriting<br>Factor 8: Test 26-Practical Judgment, Test 4-Locations<br>Factor 9: Test 29-Square Completion, Test 3-Letter Sets, Test 30-Route Planning<br>Factor 10: Test 22-Alternating Operations, Test 27-Judgment of Persons<br><br>Carroll-based factor structure:<br>Fluid Intelligence/Reasoning (Gf): Test 18-Relative Movement, Test 1-Mathematics<br>Induction/Planning (I): Test 15-Similar Rotations, Test 26-Practical Judgment, Test 29-Square Completion, Test 30-Route Planning, Test 3-Letter Sets, Test 4-Locations, Test 9-Similar Figures<br>Integration: Test 16-Paper Folding, Test 22-Alternating Operations, Test 23-Spatial Rotation,<br>Test 24-Artificial Language, Test 2-Figure Analogies (note that this factor does not appear in Carroll's model; however, it was felt that based on the descriptions of the tests, these tests could be linked to the same factor, which was labeled Integration by the authors)<br>Number Facility (N): Test 10-Addition, Test 11-Division, Test 12-Subtraction and Multiplication<br>Perceptual Speed (P): Test 13-Cancellation, Test 14-Number Checking<br>Spatial Relations (SR): Test 28-Skywriting, Test 19-Operations, Test

Abbreviation	Test	Description	Subtest-Factor Linkage[a]
			20-Directional Plotting, Test 21-Plotting<br>Verbal (Printed) Language Comprehension: Test 25-Time and Distance Estimations, Test 27-Judgment of Persons, Test 5-Vocabulary, Test 6-Verbal Reasoning<br>Visualization: Test 17-Surface Development, Test 7-Block Assembly, Test 8-Block Recognition
MAB	Multidimensional Aptitude Battery (Jackson, 1985)	The MAB is an off-the-shelf multiple-abilities test battery designed for use in personnel selection, research, and neuropsychological assessment. According to Carretta, Retzlaff, and King (1997) the MAB was developed to resemble the Wechsler Adult Intelligence Scale, although it can be administered in both individual and group settings. Carretta et al. used a computer-based testing version of the MAB, although it can also be administered using paper-and-pencil testing.	In the joint-factor-analyses with the AFOQT, the AFOQT factor-linkages described above were used and the below MAB factor-linkages were added to the model.<br>MAB-Verbal: Information (INF), Comprehension (COM), Arithmetic (ARI), Similarities (SIM), Vocabulary (VOC)<br>MAB-Performance: Digit Symbol (DIG), Picture Completion (PC), Spatial (SPA), Picture Arrangement (PA), Object Assembly (OBJ)
McGuire, Hindsman, King, and Jennings (1961)	McGuire et al. (1961)	Battery of Multiple Tests	The following factor structure, developed by Carroll (1993), was used:<br><br>F2: Gestalt Completion, Gestalt Transformation, Mechanical Reasoning/DAT, Rhymes, Step Listening, Unusual Uses<br>F3: Total Arithmetic Grd Placement, Total IQ<br>Total Language Grade Placement, Total Reading Grade Placement<br>F4: Common Situations, Consequences, Seeing Problems<br>F5: Mutilated Words, Short Words<br>F6: Clerical Speed & Accuracy/DAT, Discrimination Reaction Time, Dotting
PT	Project TALENT (American Institutes for Research, 1960a, 1960b)	Project TALENT was a large-scale longitudinal study conducted by Industrial/Organization psychologist John Flanagan and associates (1964). As part of the study, a battery of 59 mental abilities tests was administered to over 300,000 high school students. This dataset has been used in a large number of studies, primarily conducted in the 1960s-1970s (e.g., Cureton, 1968; see Campbell, 1979); however, it continues to be used in research today (e.g., Arneson, Sackett, & Beatty, 2011; Major, Johnson, & Deary, 2012; Reeve, 2001, 2004; Reeve, Meyer, & Bonacio,	Two of Major et al.'s (2012) Carroll-Horn-Cattell (CHC) factor-linkage were used: the Broad and Narrow selections. Primary loadings are shown in **bold** font and secondary loadings are shown in normal font.<br><br>**Broad Selection**<br>General Verbal Information (K0): **Vocabulary, Literature, Music, Social Studies, Biological Science, Aeronautics and Space, Art, Law, Health, Bible, Theater and Ballet, Miscellaneous, Reading Comprehension,** Mathematics, Physical Science, Memory for Words,

Abbreviation	Test	Description	Subtest-Factor Linkage[a]
		2006; Waters, 2007).	Disguised Words, Creativity<br>Math Achievement (KM): **Mathematics, Math 1, Math 2, Arithmetic Computation,** Physical Science.<br>Science Knowledge (K1): **Physical Science, Electronics, Mechanics**, Vocabulary, Biological Science, Aeronautics and Space, Health, Creativity, Mechanical Reasoning<br>English Achievement (A6): **Memory for Sentences, Memory for Words, Disguised Words, Spelling, Capitalization, Punctuation, English Usage, Effective Expression, Word Functions in Sentences,** Health, Reading Comprehension, Creativity, Abstract Reasoning, Math 2<br>Visualization (Vz): **Creativity, Mechanical Reasoning, Visualization in 2D, Visualization in 3D, Abstract Reasoning,** Word Functions in Sentences, Reading Comprehension, Math 1, Object Inspection<br>Perceptual Speed (P): **Table Reading, Clerical Checking, Object Inspection**, Disguised Words, Visualization in 2D, Arithmetic Computation<br><br>**<u>Narrow Selection</u>**<br>K0: **Vocabulary, Creativity**, Disguised Words, Reading Comprehension, Mechanical Reasoning<br>A6: **Memory for Sentences, Memory for Words, Disguised Words, Spelling, Capitalization, Punctuation, English Usage, Effective Expression, Word Functions in Sentences, Reading Comprehension,** Vocabulary, Creativity, Abstract Reasoning<br>Vz: **Mechanical Reasoning, Visualization in 2D, Visualization in 3D, Abstract Reasoning,** Creativity, Object Inspection<br>KM: **Math 1, Math 2, Arithmetic Computation**<br>P: **Table Reading, Clerical Checking, Object Inspection**, Disguised Words, Math 1, Arithmetic Computation
Reyburn and Taylor	Reyburn & Taylor (1941)	Battery of Multiple Tests	The following factor structure, developed by Carroll (1993), was used:<br><br>Crystallized Intelligence (Gc): Absurdities, Dissected Sentences, Vocabulary Test<br>Fluid Intelligence (Gf): Arithmetic Reasoning, Reasoning Test

Abbreviation	Test	Description	Subtest-Factor Linkage[a]
			Broad Visual Perception (Gv): Formboards, Porteus Mazes<br>General Memory and Learning (Gy): Match Test, Repetition of Digits/Backward, Repetition of Digits
Schipolowski, Wilhelm, and Schroeders	Schipolowski, Wilhelm, & Schroeders (2014)	Battery of Multiple Tests	Schipolowski et al.'s (2014) factor structure (termed the Publisher's factor structure) was used and a second factor model was created by rationally linking the tests to McGrew's (2005) and Carroll's (1993) factors.<br><br><u>Schipolowski et al.'s (2014) factor structure</u><br>Reading and Writing (Grw): Reading, Orthography, Writing<br>Broad Auditory Perception (Ga): Listening<br>Crystallized Intelligence (Gc): Language Usage, C-tests, Sciences, Humanities, Social Studies), Wortschatztest/WST Vocabulary, Mill Hill Vocabulary, Verbal<br>Fluid Intelligence (Gf): Numerical, Figural<br><br><u>Carroll-based factor structure</u><br>Broad Auditory Perception (Ga): Listening<br>Crystallized Intelligence (Gc): Wortschatztest/WST Vocabulary, Mill Hill Vocabulary, Verbal, Language Usage, C-tests, Sciences, Humanities, Social Studies<br>Fluid Intelligence (Gf): Numerical, Figural<br>Reading and Writing (Grw): Reading, Writing, Orthography
Thurstone	Thurstone & Thurstone (1941)	As part of a research study, Thurstone and Thurstone (1941) administered 61 mental abilities tests for use in factor analyses. Many of the tests were developed by the authors to measure a wide-range of mental abilities.	The highest salient factor loading from Table 5 of Thurstone and Thurstone (1941, p. 18-19) was used for the factor-linkages.<br><br>Induction/Reasoning (I): Directions (T12Dir), Letter Grouping (T33LetGp), Letter Series (T34LetSr), Number Patterns (T38NmPat), Pedigrees (T40Pedgr), Secret Writing (T53ScrtW)<br>Rote Memory (M): Digit Span (T11DgSpn), Figure Naming (T20FgNam), Figure Recognition (T21FgRec), First Names (T25FrstN), Word-Number Recall (T59wnumM)<br>Number (N):Addition (T3Add), Arithmetic (T5Arith), Classification (T9Classf), Multiplication (T37Mult), Reasoning (T49Rsn), Three-Higher (T56high3)

Abbreviation	Test	Description	Subtest-Factor Linkage[a]
			Perceptual (P):Mirror Reading (T7Mirr), Faces (T18Faces), Figure Grouping (T19gGrp), Identical Numbers (T30IdtNm), Identical Pictures (T31IdPix), Incomplete Words (T32IncWd), Scattered Xs (T52ScX), Mental Age (Kuhlmann-Anderson Test score [Kuhlmann & Anderson, 1927; Kuhlman, 1928) (T63KATg),<br>Space (S):Cards (T8Cards), Figures (T22Figs), Flags (T26Flags), High Numbers (T29HiNum)<br>V:Absurdities (T2Absrd), Completion (T10Compl), Disarranged Sentences (T13Disnt), Paragraph Recall (T39Recal), Proverbs (T43Prvrb), Reading Test-Vocabulary (T45Vocab), Reading Test-Sentences (T46Sent), Reading Test-Paragraphs I (T47Para1), Reading Test-Paragraphs II (T48Para2), Same or Opposite (T51SamOp)<br>Word Fluency (W):Anagrams (T4Angrm), Association (T6Asscn), First and Last Letters (T23Ltrs), First Letters (T24FrstL), Four-Letter Words (T27Four), Prefixes (T42Prefx), Rhyming Words (T50Rhym), Suffixes (T54Suffx), Synonyms (T55Syn), Word Puzzles (T60Wrdpz)<br>Dots (X1): ABC (T1ABC), Dot Counting I (T14Dcnt1), Dot Counting II (T15Dcnt2), Dot Counting III (T16Dcnt3), Dot Patterns (T17Dptrn)<br>Visual Pursuit (X2): Geometric Forms (T28Geom), Mazes I (T35Maze1), Mazes II (T36Maze2), Pursuit (T44Purs), Picture Naming (T41pixNm), Verbal Enumeration (T57vrben), Word Checking (T58wdChk),
WAIS-III; Colom, Abad, Garcia, and Juan-Espinosa (2002)	Wechsler Adult Intelligence Scale – Third Edition Spanish Version (TEA, 1998)	The WAIS-III is a cognitive ability test used to assess intelligence in both children and adults.	The WAIS-III tests were rationally linked to McGrew's (2005) and Carroll's (1993) factors by the authors.<br><br>Crystallized Intelligence (Gc): Test 1-Vocabulary, Test 5-Information, Test 6-Comprehension<br>Quantitative Knowledge (Gq): Test 3-Arithmetic<br>Short-term Memory (Gsm): Test 4-Digit Span, Test 7-Letter-number<br>Broad Visual Perception (Gv): Test 10-Block, Design, Test 11-Matrices, Test 12-Picture Arrangement, Test 14-Object Assembly, Test 2-Similarities, Test 8-Picture Completion, Test 9-Coding, Test 13-Symbol Search
WIAT-III	Wechsler Individual Achievement Test –	The WIAT-III is an achievement test that is used in a variety of settings (e.g., school, clinical, residential	The factor-linkages were obtained from the WAIT-III manual.

Abbreviation	Test	Description	Subtest-Factor Linkage[a]
	Third Edition<br>Breaux (2009)	treatment centers) to identify academic strengths and weaknesses. The WIAT-III is used to assess both children and adults.	Mathematics (M): Math Problem Solving (MPS), Numerical Operations (NO), Math Fluency Addition (MFA), Math Fluency Subtraction (MFS), Math Fluency Multiplication (MFM)<br>Oral Language (OL): Listening Comprehension (LC), Oral Expression (OE)<br>Reading (R): Early Reading Skills (ERS), Reading Comprehension (RC), Word Reading (WR), Pseudoword Decoding (PD), Oral Reading Fluency (ORF), Oral Reading Accuracy (OORA), Oral Reading Rata (ORR)<br>Written Expression (WE): Alphabet Writing Fluency (AWF), Sentence Composition (SC), Spelling (SP), Essay Composition (EC), Essay Composition Grammar and Mechanics (ECGM)<br><br>Since ORF, OORA, and ORR scores were all obtained from the same test, their errors were allowed to correlate, as were the errors for EC and ECGM.
Woodcock-Johnson III	Woodcock, McGrew, & Mather (2001).	The WJ-III is an achievement test is comprised of 22 subtests measuring a broad range of academic and cognitive abilities (e.g., reading, math, writing). The WJ-III is used with both children and adults.	Two factor-linkages from Kaufman, Reynolds, Liu, Kaufman, and McGrew (2012) were used. First, the factor-linkages for the WJ-III in Figure 2 of Kaufman et al. were used and are denoted as K12. Second, the K12 model without the correlated error terms was also run and is denoted as K12NoCE. Due to space limitations and the fact that Kaufman et al.'s paper is readily available, we do not provide the linkages here.

Note. [a]The subtest-factor linkage column indicates the assignment of subtests to factors as suggested by prior research or linkages of the subtests to Carroll's (1993) Three-Stratum Theory and McGrew's (2009) work on this topic. These linkages form the basis of the factor models. For example, for the AFOQT reported in the first row, the factor model consists of *g* and five broad factors (i.e., Math, Perceptual Speed, Spatial, Technical Knowledge, and Verbal) with the subtests loading on the broad factor(s) noted by the linkages.

Table S2. Description of datasets.

Abbreviation	Citation	Tests	Sample Size	Comments
B1001	U.S. Department of Labor (1958)	GATB	4,000	Reliabilities were obtained from Segall and Monzon (1995) and Hartigan and Wigdor (1989). Following Hartigan and Wigdor's practice for the H Markings, Two-Dimensional Space, and Speed subtests, the reliability for Form Matching was set to mean of all reliabilities below .7.

Abbreviation	Citation	Tests	Sample Size	Comments
B1002	U.S. Department of Labor (1958)	GATB	23,428	See above.
B10AY	Barto et al. (2010)	ASVAB	309,034 – 726,752	Barto et al., (2010) presented annual ASVAB data from 1989-1992 (which included the NO and CS subtests) and from 2002 to 2008 (which lacked the NO and CS subtests). The B10AY dataset was comprised of all available data on these tests from every year. Although only the 309,034 test takers from 1989-1992 took the NO and CS subtests, data from the remaining tests were based on a sample size of 726,752. The correlations were averaged using sample-size weighted Fisher-*z* transformation.
B10EY	Barto et al. (2010)	ASVAB	309,034	This dataset was an aggregation of correlation matrices from only 1989, 1990, 1991, and 1992 from Barto et al. (2010). The correlations were averaged using sample-size weighted Fisher-*z* transformation.
B90P1	Berger, Gupta, Berger, & Skinner (1990)	AFOQT	3,216	This dataset is the correlation matrix for Form P1 of the AFOQT from Berger et al. (1990).
B90P2	Berger et al. (1990)	AFOQT	2,976	This dataset is the correlation matrix for Form P2 of the AFOQT from Berger et al. (1990).
BAS11	Elliott (2011)	BAS3	1,018	The correlation matrix for the 6-18 age group was used from the technical manual. Reliabilities were not listed for ROIS, RODV, and RODS. The reliability estimate of ROIV (.76) was used for these scales, as it is conceptually similar.
C97C	Carretta et al. (1997)	AFOQT & MAB	2,233	This dataset used the multivariate range restriction corrected correlation matrix from Carretta et al. (1997).
C97U	Carretta et al. (1997)	AFOQT & MAB	2,233	This dataset used the uncorrected/observed correlation matrix from Carretta et al. (1997). AFOQT reliabilities were obtained from Berger et al. (1990). Reliability information on the MAB could not be located. Therefore, Charter's (2003) reliability generalization values for intelligence and aptitude tests were used.
COGAT	Lohman & Hagen (2002)	CogAt	103,044	No internal consistency estimates were provided for individual tests; however, the manual reports an average Kuder-Richardson reliability estimate for the Verbal, Quantitative, and Nonverbal test batteries as .95, .94, and .95, respectively.

Abbreviation	Citation	Tests	Sample Size	Comments
COL02	Colom (2002)	WAIS	1,369	No internal consistency estimates were provided for individual tests; internal consistencies from Charter (2003) were used.
Combined	Liepmann et al. (2001)	IST	4,102	This dataset used the correlations obtained from test takers in both the United Kingdom and Germany. The correlations were averaged using sample-size weighted Fisher-*z* transformation.
DASM	Elliott (1990)	DAS	2,400	This dataset used the correlation matrix published in the DAS manual.
DATM	Psychological Corporation (1991)	DAT & ASVAB	1338	It was assumed that *M*=50 and *SD*=10 for all tests (the ASVAB was normed to yield these values).<br><br>DAT Manual; ASVAB reliabilities for WK, AR, MK, EI, PC, and MC from Table 2 Brown et al. (2006); ASVAB reliabilities for NO, GS, AS, CS from Table 7 Welsh, Kucinkas, and Curran (1990) pp. 26-27.
DETT00	Detterman (1985)	Research-based test battery, related to ASVAB	502	
DRAS10	Drasgow, Ney, Carretta, and Ree (2010)	AFOQT	12,511	
EASM	Ruch et al. (1994)	EAS	1,406	
German	Liepmann et al. (2001)	IST	2,208	This dataset used the correlations obtained from test takers in the Germany.
GUIL32A	Guilford & Lacey (1947)	Research-based Aviation Classification Test	1,024	No internal consistency estimates were provided for individual tests; therefore, internal consistencies from Charter (2003) were used.
GUST84	Gustafsson (1984)	Research-based test battery	981	No internal consistency estimates were provided for several scales; therefore, internal consistencies from Gustaffson (1981) were used. Reliability for Ravens scales was calculated using the Spearman-Brown Prophecy formula to obtain split half reliability; the same process was

Abbreviation	Citation	Tests	Sample Size	Comments
				used to compute estimates of reliability for MF, O, and CR.
JNB08	Johnson, Nijenhuis, and Bouchard (2008).	Battery of Multiple Tests	500	No internal consistency estimates were provided for individual tests; therefore, internal consistencies from Charter (2003) were used.
K77	Kettner (1977)	DAT & ASVAB & GATB	616 + 616	Kettner (1977) administered the ASVAB to 1,232 students. Of these students, 616 took the DAT and the remaining 616 took the GATB. Kettner reports correlations separately for male and female 9th, 10th, 11th, and 12th graders. The correlations were averaged using sample-size weighted Fisher-*z* transformation.<br><br>Welsh, Kucinkas, & Curran's (1990) reliability estimate for combined AI/SI was used for both the AI and SI subtests. Reliabilities for GI, AD, SP, and GB are the averages of the reliabilities on the other subtests.
KABC-M	Kaufman & Kaufman (2004)	KABC	975	The *M* and *SD* for AD and RD could not be located, therefore these were set to 10 and 3, respectively.
KAIT-M	Kaufman & Kaufman (1993)	KAIT	2,000	Since the split-half reliabilities were highest in nearly all cases, they were used as the reliability estimates. The manual does not list the *M* or *SD* for the last two subtests (i.e., the delayed recall tests); however, it does state that *M*s and *SD*s standardization are fixed to 10 and 3 for each age group within standardization sample; therefore we assumed a *M* of 10 and a *SD* of 3.
KASS83	Kass et al. (1983)	ASVAB	98,689	
LUCAS	Lucas & French (1953)	Research-based Naval test battery	666	No internal consistency estimates were provided for individual tests; internal consistencies from Charter (2003) were used.
MCGU01	McGuire et al. (1961)	Battery of Multiple Tests	1,242	
NLSY79	U.S. Bureau of Labor Statistics, U.S. Department of Labor. (2012a)	ASVAB	11878	See above.

Abbreviation	Citation	Tests	Sample Size	Comments
NLSY97	U.S. Bureau of Labor Statistics, U.S. Department of Labor. (2012b)	ASVAB	6,965	See above.
PT-Broad & PT-Narw.	American Institutes for Research. (1960b).	Research-based test battery	321,589	The base-year dataset from Project TALENT (American Institutes for Research, 1960a/b) was used. More information on the dataset and study can be found in Flanagan et al. (1961, 1964, Wise, McLaughlin, & Steel, 1979).<br><br>The "Broad" dataset used Major et al.'s (2013) "broad" selection of tests and the "Narw." dataset used their "narrow" selection; both datasets had the same cases and only differed in terms of the number of indicator variables used in the analyses.<br><br>Four types of reliability coefficients were used. First, Major et al. (2013) report reliability coefficients for many of the Project TALENT tests. Second, the study documentation reports KR-20 (which is equivalent to Coefficient Alpha) for other tests. Third, some of the test reliabilities were KR-21 coefficients, which is an anachronistic approximation of KR-20. Fourth, Charter's (2003) reliability estimates for intelligence and aptitude tests were used for any reliabilities that could not be obtained.
RE90	Ree & Earles (1990)	ASVAB	9,173	ASVAB reliabilities for WK, AR, MK, EI, PC, and MC were obtained from Table 2 of Brown et al. (2006); ASVAB reliabilities for NO, GS, AS, CS from Table 7 of Welsh, Kucinkas, & Curran (1990, pp. 26-27). A mean of 50 and standard deviation of 10 was assumed (the ASVAB is normed to produce these values).
REE82	Ree, Mullins, Mathews, and Massey (1982)	ASVAB	2,620	Only the correlations matrix and sample size were provided. Internal consistency estimates, means, and standard deviations for individual tests were taken from Ree et al. (1982).
REYB01	Reyburn & Taylor (1941)	Battery of Multiple Tests	1,497	No internal consistency estimates were provided for individual tests; internal consistencies from Charter (2003) were used.
SCHIP14	Schipolowski, Wilhelm, &	Battery of	1,957	The sample sizes varied by subtest; the median sample size was used in

Abbreviation	Citation	Tests	Sample Size	Comments
	Schroeders (2014)	Multiple Tests		the analysis.
SEG01A	Sawin, Earles, Goff, & Chaiken (2001)	LAMP & ASVAB	9,325	This dataset is based on the first sample reported by Sawin et al. (2001). It was composed of Air Force basic recruits. Sawin et al. provide standard deviations but not means; therefore the mean was arbitrarily set to 50.
SEG01B	Sawin et al. (2001)	LAMP & ASVAB	2,270	This dataset is based on the second sample reported by Swain et al. (2001). It was composed of trainees in a security police school within the U.S. Air Force.
T41	Thurstone & Thurstone (1941)	Research-based test battery	710	Charter's (2003) reliability for intelligence and aptitude tests was used.
TEAC14	Teachout, Ree, Barto, Carretta, and King (2014) et al. (2014)	MAB-II and MicroCog	10,612	No internal consistency estimates were provided for individual tests; therefore, internal consistencies from Charter (2003) were used.
THOR21	Thorndike (1936)	George Washington Mental Alertness and Social Intelligence Test	500	
UK	Liepmann et al. (2001)	IST	1,894	This dataset used the correlations obtained from test takers in the United Kingdom.
WIATM	Breaux (2009)	WIAT	1,375	
WJIIIM	McGrew & Woodcock (2001).	WJ-III	6,189	McGrew and Woodcock (2001) provide separate correlation matrices for a variety of age groups. The correlations for test takers who were age 6 and above were averaged using sample-size weighted Fisher-*z* transformation. The sample sizes for the subtest correlations are different, not only for the different age groups, but also for different subtests within each age group; only a single number was provided for each test. For this reason, we used the median sample size across the

Abbreviation	Citation	Tests	Sample Size	Comments
				variables for each group. Further note that some of the younger age groups only completed a subset of the test battery, thus when we averaged across the groups, some correlation coefficients were based on large sample sizes than others. Thus, the total averaged correlation matrix had sample sizes ranging from 6,189 to 7,283. We decided to use the minimum value (6,189) as the final sample size for the correlation matrix. This value is lower than the sample size of 8,818 given in the manual.

Table S3. Fit statistics for best fitting models for each dataset.

Dataset	Model	2 Ind.		Higher-Order								Comparison			Bifactor							
			Notes	CFI	TLI	NFI	RMSEA	AIC	χ^2	*df*	*p*	$\Delta\chi^2$	*df*	*p*	CFI	TLI	NFI	RMSEA	AIC	χ^2	*df*	*p*
**ASVAB**																						
B10AY	RC94	Baseln	a	.851	.797	.851	.141	274454	274410	33	<.001											
B10AY	RC94	Mixed	b	.840	.782	.840	.146	294857	294813	33	<.001	174185	6	<.001	**.935**	**.891**	**.935**	**.103**	**120683**	120627	27	<.001
B10AY	RC94	CE	b	.840	.782	.840	.146	294857	294813	33	<.001	174185	6	<.001	**.935**	**.891**	**.935**	**.103**	**120683**	120627	27	<.001
B10AY	RC94	Drop		.807	.744	.807	.159	357192	357150	34	<.001	140156	6	<.001	**.883**	**.811**	**.883**	**.136**	**217048**	216994	28	<.001
B10AY	RC94	fix	a	.777	.714	.777	.168	411761	411721	35	<.001	143660	7	<.001	**.855**	**.767**	**.855**	**.151**	**268115**	268061	28	<.001
B10EY	RC94	Mixed	a	.847	.791	.847	.133	179590	179546	33	<.001	43671	6	<.001	**.926**	**.877**	**.926**	**.110**	**135931**	135875	27	<.001
B10EY	RC94	CE	a	.847	.791	.847	.133	179590	179546	33	<.001	43671	6	<.001	**.926**	**.877**	**.926**	**.110**	**135931**	135875	27	<.001
B10EY	RC94	Drop	a	.795	.729	.795	.151	240450	240408	34	<.001	23414	6	<.001	**.883**	**.811**	**.883**	**.136**	**217048**	216994	28	<.001
B10EY	RC94	fix	a	.761	.692	.761	.161	281179	281139	35	<.001	13078	7	<.001	**.855**	**.767**	**.855**	**.151**	**268115**	268061	28	<.001
K82	RC94	Mixed	a, c	.894	.859	.894	.155	80881	80839	34	<.001	33559	5	<.001	**.938**	**.904**	**.938**	**.128**	**47332**	47280	29	<.001
K82	RC94	Mixed	d												.974	.955	.974	.088	19897	19839	26	<.001
K82	RC94	CE	a	.856	.809	.856	.181	109696	109654	34	<.001	77450	6	<.001	**.958**	**.932**	**.958**	**.108**	**32258**	32204	28	<.001
K82	RC94	CE	d												.974	.955	.974	.088	19897	19839	26	<.001
K82	RC94	Drop		.866	.823	.866	.174	101884	101842	34	<.001	51245	7	<.001	**.933**	**.889**	**.933**	**.138**	**50653**	50597	27	<.001
K82	RC94	fix	a, e	.845	.807	.845	.182	117601	117563	36	<.001	78126	7	<.001	**.948**	**.92**	**.948**	**.117**	**39489**	39437	29	<.001
K82	RC94	fix	d												.964	.941	.964	.101	27174	27118	27	<.001
NLSY79	RC94	Baseln	f	.911	.879	.911	.156	9599	9555	33	<.001											

Dataset	Model	2 Ind.		Higher-Order								Comparison			Bifactor							
			Notes	CFI	TLI	NFI	RMSEA	AIC	χ^2	*df*	*p*	$\Delta\chi^2$	*df*	*p*	CFI	TLI	NFI	RMSEA	AIC	χ^2	*df*	*p*
NLSY79	RC94	Mixed	[g]												.978	.962	.978	.088	2469	2411	26	<.001
NLSY79	RC94	CE	[a, h]	.887	.850	.887	.173	12225	12183	34	<.001	8169	6	<.001	**.963**	**.940**	**.963**	**.109**	**4067**	4013	28	<.001
NLSY79	RC94	Drop	[a, i]	.846	.796	.845	.203	16666	16624	34	<.001	6379	6	<.001	**.905**	**.847**	**.905**	**.175**	**10298**	10244	28	<.001
NLSY79	RC94	fix	[a]	.883	.854	.883	.171	12645	12607	36	<.001	8519	7	<.001	**.962**	**.941**	**.962**	**.109**	**4140**	4088	29	<.001
NLSY97	RC94M	Mixed	[a]	.963	.953	.961	.055	1181	1129	52	<.001	526	8	<.001	**.981**	**.971**	**.979**	**.043**	**671**	603	44	<.001
NLSY97	RC94M	Mixed	[d]												.981	.971	.979	.043	671	603	44	<.001
NLSY97	RC94M	CE	[a]	.962	.953	.960	.055	1217	1167	53	<.001	564	9	<.001	**.981**	**.971**	**.979**	**.043**	**671**	603	44	<.001
NLSY97	RC94M	Drop	[a]	.962	.953	.960	.055	1217	1167	53	<.001	526	8	<.001	**.980**	**.970**	**.978**	**.044**	**707**	641	45	<.001
NLSY97	RC94M	fix	[a]	.899	.876	.897	.089	3061	3013	54	<.001	770	9	<.001	**.925**	**.890**	**.923**	**.084**	**2309**	2243	45	<.001
R82	RC94	Baseln	[g]	.814	.753	.813	.218	14847	14805	34	<.001											
R82	RC94	Mixed	[j]	.787	.709	.787	.236	16961	16917	33	<.001	8484	6	<.001	**.894**	**.823**	**.894**	**.184**	**8488**	8432	27	<.001
R82	RC94	CE	[j]	.787	.709	.787	.236	16961	16917	33	<.001	8484	6	<.001	**.894**	**.823**	**.894**	**.184**	**8489**	8433	27	<.001
R82	RC94	Drop	[j, k]	.771	.706	.771	.237	18180	18140	35	<.001	8536	6	<.001	**.879**	**.812**	**.879**	**.190**	**9656**	9604	29	<.001
R82	RC94	fix	[j]	.786	.725	.786	.230	17013	16973	35	<.001	8533	7	<.001	**.894**	**.829**	**.894**	**.181**	**8494**	8440	28	<.001
RE90	RC94	Mixed	[a]	.887	.846	.887	.172	9021	8977	33	<.001											
RE90	RC94	Mixed	[d]												.971	.95	.971	.098	2356	2298	26	<.001
RE90	RC94	CE	[a]	.887	.846	.887	.172	9021	8977	33	<.001	6679	7	<.001	**.971**	**.950**	**.971**	**.098**	**2356**	2298	26	<.001
RE90	RC94	Drop	[l]	.809	.747	.809	.220	15191	15149	34	<.001	10318	7	<.001	**.939**	**.899**	**.939**	**.139**	**4887**	4831	27	<.001
RE90	RC94	fix	[a]	.882	.848	.882	.171	9431	9391	35	<.001	7076	8	<.001	**.971**	**.952**	**.971**	**.096**	**2371**	2315	27	<.001
**AFOQT**																						
B90P1	CR95	Baseln		.909	.884	.906	.094	2863	2779	94	<.001											
B90P1	CR95		[m]	.908	.884	.905	.094	2877	2795	95	<.001	730	11	<.001	**.933**	**.904**	**.930**	**.086**	**2169**	2065	84	<.001
B90P2	CR95			.923	.901	.920	.088	2367	2283	94	<.001	584	11	<.001	**.943**	**.918**	**.940**	**.081**	**1805**	1699	83	<.001
C97U	CR95			.901	**.873**	.894	**.078**	1457	1373	94	<.001	146	11	<.001	**.911**	.872	**.906**	.079	**1333**	1227	83	<.001
C97C	CR95			.940	.924	.936	.075	1367	1283	94	<.001	353	11	<.001	**.958**	**.939**	**.954**	**.068**	**1036**	930	83	<.001
D10	CR95	Baseln	[n]	.835	.778	.834	.143	10559	10509	41	<.001											
D10	CR95	Mixed	[d]												.921	.880	.921	.105	5082	5022	36	<.001
D10	CR95	Mixed	[n, o]	.807	.747	.806	.153	12329	12281	42	<.001	6963	4	<.001	**.917**	**.879**	**.916**	**.105**	**5374**	5318	38	<.001
D10	CR95	CE	[m, o]	.807	.747	.806	.153	12329	12281	42	<.001	7239	5	<.001	**.921**	**.882**	**.920**	**.104**	**5100**	5042	37	<.001
D10	CR95	Drop	[p]	.740	.668	.740	.175	16532	16486	43	<.001	7146	4	<.001	**.853**	**.793**	**.853**	**.138**	**9393**	9339	39	<.001

Dataset	Model	2 Ind.		Higher-Order								Comparison			Bifactor							
			Notes	CFI	TLI	NFI	RMSEA	AIC	χ^2	*df*	*p*	$\Delta\chi^2$	*df*	*p*	CFI	TLI	NFI	RMSEA	AIC	χ^2	*df*	*p*
D10	CR95	fix	q	.544	.455	.544	.224	28932	28892	46	<.001	10945	6	<.001	**.717**	**.611**	**.717**	**.189**	**17998**	17946	40	<.001
**AFOQT & MAB**																						
C97U	CR95			.814	.789	.803	.075	4038	3908	286	<.001	381	19	<.001	**.833**	**.797**	**.822**	**.074**	**3695**	3527	267	<.001
C97C	CR95			.866	.848	.858	.079	4382	4252	286	<.001	788	19	<.001	**.892**	**.869**	**.884**	**.073**	**3631**	3463	267	<.001
**BAS3**																						
BAS3M	CHC	Baseln		.764	.724	.753	.122	2204	2124	131	<.001											
BAS3M	CHC	Mixed	r	.690	.640	.680	.140	2833	2755	132	<.001	1566	12	<.001	**.873**	**.839**	**.862**	**.094**	**1291**	1189	120	<.001
BAS3M	CHC	CE	r,s	.722	.677	.711	.132	2561	2483	132	<.001	1374	12	<.001	**.883**	**.851**	**.871**	**.090**	**1211**	1109	120	<.001
BAS3M	CHC	Drop	t	.734	.694	.723	.129	2459	2383	133	<.001	1043	12	<.001	**.856**	**.818**	**.844**	**.100**	**1440**	1340	121	<.001
BAS3M	CHC	fix		.553	.486	.545	.167	3986	3910	133	<.001	2561	13	<.001	**.855**	**.815**	**.843**	**.100**	**1451**	1349	120	<.001
**CogAt**																						
CogAtM	CogAtM			.994	**.991**	.994	**.041**	4182	4140	24	<.001	797	6	<.001	**.995**	.990	**.995**	.042	**3397**	3343	18	<.001
**Colom (2002)**																						
C02	C02	Baseln		.939	.925	.935	.100	1143	1081	74	<.001											
C02	C02	Mixed	u	.922	.906	.918	.112	1412	1352	75	<.001	412	9	<.001	**.947**	**.927**	**.943**	**.098**	**1017**	939	66	<.001
C02	C02	CE		.939	.925	.935	.100	1143	1081	74	<.001	283	9	<.001	**.955**	**.938**	**.952**	**.091**	**878**	798	65	<.001
C02	C02	Drop	u	.914	.897	.910	.117	1554	1496	76	<.001	275	9	<.001	**.930**	**.905**	**.926**	**.112**	**1297**	1221	67	<.001
C02	C02	fix	v	.918	.903	.914	.113	1482	1426	77	<.001	494	10	<.001	**.947**	**.928**	**.944**	**.097**	**1009**	933	67	<.001
**DAT**																						
DATM	DATM	Mixed		.937	.901	.934	.134	488	452	18	<.001	164	3	<.001	**.960**	**.925**	**.958**	**.117**	**330**	288	15	<.001
DATM	DATM	CE		.937	.901	.934	.134	488	452	18	<.001	164	3	<.001	**.960**	**.925**	**.958**	**.117**	**330**	288	15	<.001
DATM	DATM	Drop		.918	.880	.916	.148	610	576	19	<.001	120	3	<.001	**.936**	**.887**	**.934**	**.143**	**496**	456	16	<.001
DATM	DATM	fix		.691	**.567**	.689	**.281**	2163	2131	20	<.001	161	3	<.001	**.714**	.529	**.713**	.293	**2009**	1971	17	<.001
K77	RE90	Mixed		.921	.877	.916	.148	298	262	18	<.001	202	4	<.001	**.985**	**.970**	**.981**	**.073**	104	60	14	<.001
K77	RE90	CE		.921	.877	.916	.148	298	262	18	<.001	202	4	<.001	**.985**	**.970**	**.981**	**.073**	104	60	14	<.001
K77	RE90	Drop		.919	.880	.913	.146	304	270	19	<.001	180	4	<.001	**.976**	**.955**	**.971**	**.090**	132	90	15	<.001
K77	RE90	fix		.000	-.905	-.558	.584	4874	4848	23	<.001	4348	5	<.001	**.844**	**.757**	**.839**	**.209**	**536**	500	18	<.001
**DAT & ASVAB**																						
DATM	DATM		w	.889	.870	.883	.112	2427	2347	131	<.001	347	10	<.001	**.906**	**.881**	**.901**	**.108**	**2100**	2000	121	<.001
K77	DATM	Baseln		.798	.771	.784	.132	2250	2158	185	<.001											

Dataset	Model	2 Ind.		Higher-Order								Comparison			Bifactor							
			Notes	CFI	TLI	NFI	RMSEA	AIC	χ^2	*df*	*p*	$\Delta\chi^2$	*df*	*p*	CFI	TLI	NFI	RMSEA	AIC	χ^2	*df*	*p*
K77	DATM		[x]	.797	.771	.783	.132	2259	2169	186	<.001	804	17	<.001	**.878**	**.848**	**.863**	**.107**	**1490**	1366	169	<.001
**DAS**																						
DASM	DASM	Baseln	[y]	.897	.875	.893	.094	1987	1921	87	<.001											
DASM	DASM		[z]	.842	.811	.838	.115	2964	2900	88	.000	1432	8	<.001	**.922**	**.898**	**.918**	**.085**	**1548**	**1468**	80	<.001
**Determan (2000)**																						
D00	D00	Mixed		.678	.649	.602	.069	1578	1438	426	<.001	84	20	<.001	**.699**	**.655**	**.625**	**.068**	**1534**	1354	406	<.001
D00	D00	CE		.678	.649	.602	.069	1578	1438	426	<.001	84	20	<.001	**.699**	**.655**	**.625**	**.068**	**1534**	1354	406	<.001
D00	D00	Drop		.641	.611	.568	.073	1693	1559	429	<.001	75	20	<.001	**.658**	**.612**	**.589**	**.072**	**1658**	1484	409	<.001
D00	D00	fix		.000	-.207	-.096	.128	4088	3960	432	<.001	980	23	<.001	**.183**	**.071**	**.175**	**.112**	**3154**	2980	409	<.001
**EAS**																						
EASM	CHC	Baseln		.849	.787	.839	.093	463	417	32	<.001											
EASM	CHC	Mixed	[aa]	.622	.485	.616	.144	1037	993	33	<.001	589	4	<.001	**.852**	**.771**	**.844**	**.096**	**457**	405	29	<.001
EASM	CHC	CE		.849	.787	.839	.093	463	417	32	<.001	58	4	<.001	**.870**	**.791**	**.861**	**.092**	**413**	359	28	<.001
EASM	CHC	Drop	[aa]	.592	.460	.586	.147	1113	1071	34	<.001	573	4	<.001	**.816**	**.724**	**.807**	**.105**	**548**	498	30	<.001
EASM	CHC	fix	[ab]	.000	-.878	-.449	.275	3787	3747	35	<.001	2958	5	<.001	**.701**	**.552**	**.695**	**.134**	**840**	790	30	<.001
**GATB**																						
B1001	CHC	Mixed		.820	.783	.818	.133	6295	6229	87	<.001	1410	9	<.001	**.861**	**.813**	**.859**	**.123**	**4903**	4819	78	<.001
B1001	CHC	CE		.820	.783	.818	.133	6295	6229	87	<.001	1410	9	<.001	**.861**	**.813**	**.859**	**.123**	**4903**	4819	78	<.001
B1001	CHC	Drop		.780	.738	.778	.146	7647	7583	88	<.001	1137	9	<.001	**.813**	**.752**	**.812**	**.142**	**6529**	6447	79	<.001
B1001	CHC	fix		.816	.783	.814	.133	6424	6362	89	<.001	1542	10	<.001	**.861**	**.815**	**.859**	**.123**	**4902**	4820	79	<.001
B1002	CHC	Baseln	[ac]	.855	.786	.855	.165	12101	12067	19	<.001											
B1002	CHC		[ad]	.801	.735	.801	.183	16542	16512	21	<.001	6182	4	<.001	**.876**	**.795**	**.875**	**.161**	**10368**	10330	17	<.001
B1002	CHC		[ae]												.894	.803	.894	.158	8812	8770	15	<.001
K77	P93	Mixed		.910	.860	.902	.125	228	192	18	<.001	54	4	<.001	**.936**	**.872**	**.930**	**.120**	**182**	138	14	<.001
K77	P93	CE		.910	.860	.902	.125	228	192	18	<.001	54	4	<.001	**.936**	**.872**	**.930**	**.120**	**182**	138	14	<.001
K77	P93	Drop		.909	.866	.901	.123	228	194	19	<.001	45	4	<.001	**.930**	**.870**	**.924**	**.121**	**191**	149	15	<.001
K77	P93	fix		.876	.826	.867	.140	292	260	20	<.001	111	5	<.001	**.930**	**.870**	**.924**	**.121**	**192**	150	15	<.001
**GATB & ASVAB**																						
K77	P93	Baseln	[af]	.807	.774	.788	.114	1712	1610	180	<.001											
K77	P93		[ag]												.882	.849	.864	.093	1170	1036	164	<.001

Dataset	Model	2 Ind.		Higher-Order								Comparison			Bifactor							
			Notes	CFI	TLI	NFI	RMSEA	AIC	χ^2	*df*	*p*	$\Delta\chi^2$	*df*	*p*	CFI	TLI	NFI	RMSEA	AIC	χ^2	*df*	*p*
K77	P93		[ah]	.786	.751	.768	.119	1866	1766	181	<.001	727	16	<.001	**.882**	**.850**	**.863**	**.093**	**1171**	1039	165	<.001
**Guilford (1932)**																						
G32	CHC	Mixed		.924	.905	.899	.051	332	268	73	<.001	34	9	<.001	**.933**	.905	**.911**	.051	**316**	234	64	<.001
G32	CHC	CE		.793	.748	.771	.083	663	603	75	<.001	370	11	<.001	**.933**	**.905**	**.911**	**.051**	**316**	234	64	<.001
G32	CHC	Drop		.372	.248	.365	.143	1734	1676	76	<.001	1364	11	<.001	**.903**	**.864**	**.882**	**.061**	**392**	312	65	<.001
G32	CHC	fix		.470	.357	.460	.133	1485	1425	75	<.001	934	10	<.001	**.833**	**.766**	**.814**	**.080**	**571**	491	65	<.001
**Gustafsson (1984)**																						
G84	G84	Mixed		.916	.900	.901	.073	1098	1000	161	<.001	124	4	<.001	**.928**	**.912**	**.914**	**.068**	**982**	876	157	<.001
G84	G84	CE		.916	.900	.901	.073	1098	1000	161	<.001	124	4	<.001	**.928**	**.912**	**.914**	**.068**	**982**	876	157	<.001
G84	G84	Drop		.673	.631	.663	.140	3499	3415	168	<.001	110	4	<.001	**.684**	**.634**	**.674**	.140	**3398**	3306	164	<.001
G84	G84	fix		.791	.773	.777	.110	2328	2258	175	<.001	695	11	<.001	**.859**	**.837**	**.846**	**.093**	**1655**	1563	164	<.001
**IST**																						
German	IST-M			.980	.970	.977	**.050**	199	157	24	<.001	37	6	<.001	**.985**	.970	**.982**	.051	**174**	120	18	<.001
UK	IST-M			.976	.964	.973	.060	232	190	24	<.001	63	6	<.001	**.984**	**.969**	**.982**	**.057**	**181**	127	18	<.001
Combined	IST-M			.980	.969	.978	.053	341	299	24	<.001	74	6	<.001	**.985**	.969	**.983**	.053	**279**	225	18	<.001
**Johnson et al. (2008)**																						
J08	J08M	Baseln		.682	.662	.624	.081	4388	4174	974	<.001											
J08	J08M	Mixed		.654	.633	.598	.085	4668	4458	976	<.001	425	21	<.001	**.694**	**.668**	**.636**	**.080**	**4285**	4033	955	<.001
J08	J08M	CE		.654	.633	.598	.085	4668	4458	976	<.001	425	21	<.001	**.694**	**.668**	**.636**	**.080**	**4285**	4033	955	<.001
J08	J08M	Drop		.629	.609	.575	.087	4915	4717	982	<.001	398	21	<.001	**.666**	**.640**	**.611**	**.084**	**4559**	4319	961	<.001
J08	J08M	fix		.233	.196	.216	.125	8892	8704	987	<.001	1492	27	<.001	**.379**	**.330**	**.350**	**.114**	**7454**	7212	960	<.001
**KABC**																						
KABC-M	KABCM	Mixed	[ai]	.943	.931	.932	.069	629	555	99	<.001	113	10	<.001	**.956**	**.941**	**.946**	**.064**	**537**	443	89	<.001
KABC-M	KABCM	Mixed	[d]												.974	.965	.964	.049	392	296	88	<.001
KABC-M	KABCM	CE		.962	.953	.950	.057	482	406	98	<.001	110	10	<.001	**.974**	**.965**	**.964**	**.049**	**392**	296	88	<.001
KABC-M	KABCM	Drop		.962	.954	.950	.056	480	406	99	<.001	110	10	<.001	**.974**	**.965**	**.964**	**.049**	**390**	296	89	<.001
KABC-M	KABCM	fix		.845	.813	.835	.114	1427	1355	100	<.001	833	11	<.001	**.946**	**.928**	**.936**	**.071**	**617**	523	89	<.001
**KAIT**																						
KAIT-M	CHC	Mixed		.919	.886	.917	.124	1062	1016	32	<.001	531	5	<.001	**.962**	**.937**	**.960**	**.092**	**542**	486	27	<.001
KAIT-M	CHC	CE		.919	.886	.917	.124	1062	1016	32	<.001	531	5	<.001	**.962**	**.937**	**.960**	**.092**	**542**	486	27	<.001

**Dataset**	**Model**	**2 Ind.**		**Higher-Order**								**Comparison**			**Bifactor**							
			**Notes**	**CFI**	**TLI**	**NFI**	**RMSEA**	**AIC**	χ^2	*df*	*p*	$\Delta\chi^2$	*df*	*p*	**CFI**	**TLI**	**NFI**	**RMSEA**	**AIC**	χ^2	*df*	*p*
KAIT-M	CHC	Drop		.919	.889	.916	.122	1064	1020	33	<.001	503	5	<.001	**.960**	**.935**	**.958**	**.093**	**571**	517	28	<.001
KAIT-M	CHC	fix		.688	.587	.686	.236	3867	3825	34	<.001	2849	6	<.001	**.922**	**.874**	**.920**	**.130**	**1030**	976	28	<.001
**LAMP**																						
SEG01A	RE90	Baseln	aj	.947	.931	.945	.051	1322	1268	51	<.001											
SEG01A	RE90		ak	.649	.554	.647	.129	8131	8079	52	<.001	7234	8	<.001	**.965**	**.947**	**.963**	**.044**	**913**	845	44	<.001
SEG01A	RE90		al												.967	.950	.965	.043	860	790	43	<.001
SEG01B	RE90	Baseln	am	.942	.925	.934	.052	414	360	51	<.001											
SEG01B	RE90		an								<.001				.968	.950	.960	.042	286	216	43	<.001
SEG01B	RE90		ao	.001	-.274	-.001	.213	5478	5426	52	<.001	4773	8	<.001	**.886**	**.829**	**.880**	**.078**	**721**	653	44	<.001
**LAMP & ASVAB**																						
SEG01A	RE90	Baseln		.818	.792	.816	.077	11352	11250	202	<.001											
SEG01A	RE90	Mixed	ap	.767	.735	.764	.087	14479	14379	203	<.001	4883	14	<.001	**.847**	**.813**	**.844**	**.073**	**9625**	9497	189	<.001
SEG01A	RE90	CE	ap	.767	.735	.764	.087	14479	14379	203	<.001	4883	14	<.001	**.847**	**.813**	**.844**	**.073**	**9625**	9497	189	<.001
SEG01A	RE90	Drop	ap	.713	.675	.711	.096	17732	17634	204	<.001	4884	14	<.001	**.793**	**.749**	**.791**	**.084**	**12876**	12750	190	<.001
SEG01A	RE90	fix	ap	.743	.711	.741	.090	15904	15808	205	<.001	4883	15	<.001	**.823**	**.785**	**.821**	**.078**	**11052**	10926	190	<.001
SEG01B	RE90	Baseln		.804	.776	.794	.081	3284	3182	202	<.001											
SEG01B	RE90	Mixed	ap	.753	.719	.744	.090	4046	3946	203	<.001	1289	14	<.001	**.837**	**.801**	**.828**	**.076**	**2786**	2658	189	<.001
SEG01B	RE90	CE	ap	.753	.719	.744	.090	4046	3946	203	<.001	1289	14	<.001	**.837**	**.801**	**.828**	**.076**	**2786**	2658	189	<.001
SEG01B	RE90	Drop		.743	.707	.733	.092	4212	4112	203	<.001	522	14	<.001	**.776**	**.726**	**.767**	**.089**	**3718**	3590	189	<.001
SEG01B	RE90	fix	ap	.737	.703	.727	.093	4300	4204	205	<.001	1289	15	<.001	**.820**	**.782**	**.811**	**.080**	**3042**	2916	190	<.001
**Lucas & French (1953)**																						
LF53	LF53	Baseln	aq	.870	.858	.818	.054	1316	1178	396	<.001											
LF53	LF53	Mixed	ar												.893	.878	.842	.050	1193	1023	380	<.001
LF53	LF53	Mixed	as	.851	.837	.800	.058	1432	1296	397	<.001	273	16	<.001	**.894**	**.878**	**.842**	**.050**	**1191**	1023	381	<.001
LF53	LF53	CE	aq	.851	.837	.800	.058	1432	1296	397	<.001	273	16	<.001	**.894**	**.878**	**.842**	**.050**	**1191**	1023	381	<.001
LF53	LF53	Drop	at	.820	.804	.770	.064	1617	1489	401	<.001	274	16	<.001	**.862**	**.845**	**.812**	**.057**	**1375**	1215	385	<.001
LF53	LF53	fix	at	.000	-.093	-.012	.151	6665	6545	405	<.001	1863	20	<.001	**.288**	**.195**	**.276**	**.130**	**4842**	4682	385	<.001
LF53	CHC	Baseln	aq	.847	.833	.796	.059	1456	1322	398	<.001											
LF53	CHC	Mixed	#au												.871	.851	.821	.056	1331	1157	378	<.001
LF53	CHC	Mixed	#av	.811	.794	.762	.066	1670	1538	399	<.001	379	20	<.001	**.871**	**.852**	**.821**	**.056**	**1331**	1159	379	<.001

Dataset	Model	2 Ind.		Higher-Order								Comparison			Bifactor							
			Notes	CFI	TLI	NFI	RMSEA	AIC	χ^2	*df*	*p*	$\Delta\chi^2$	*df*	*p*	CFI	TLI	NFI	RMSEA	AIC	χ^2	*df*	*p*
LF53	CHC	CE	#av	.811	.794	.762	.066	1670	1538	399	<.001	379	20	<.001	**.871**	**.852**	**.821**	**.056**	**1331**	1159	379	<.001
LF53	CHC	Drop	#av	.780	.762	.733	.070	1854	1726	401	<.001	382	20	<.001	**.840**	**.818**	**.792**	**.062**	**1512**	1344	381	<.001
LF53	CHC	fix	aq	.609	.577	.574	.094	2884	2758	402	<.001	532	22	<.001	**.694**	**.650**	**.656**	**.085**	**2396**	2226	380	<.001
**MAB & MicroCog**																						
T14U	T14U	Baseln		.744	.691	.742	.097	8920	8854	87	<.001											
T14U	T14U		aw	.613	.538	.611	.119	13392	13328	88	<.001	8743	12	<.001	**.868**	**.818**	**.866**	**.075**	**4673**	4585	76	<.001
T14C	T14C			.736	.681	.735	.158	23179	23113	87	<.001	3680	12	<.001	**.778**	**.689**	**.777**	**.156**	**19524**	19434	75	<.001
**McGuire et al. (1961)**																						
M61	M61	Mixed		.927	.914	.915	.066	920	838	130	<.001	105	12	<.001	**.936**	**.917**	**.925**	**.065**	**839**	733	118	<.001
M61	M61	CE		.927	.914	.915	.066	920	838	130	<.001	105	12	<.001	**.936**	**.917**	**.925**	**.065**	**839**	733	118	<.001
M61	M61	Drop		.922	.909	.910	.068	968	888	131	<.001	102	12	<.001	**.931**	**.911**	**.920**	**.067**	**890**	786	119	<.001
M61	M61	fix		.722	.678	.713	.128	2896	2818	132	<.001	1103	13	<.001	**.835**	**.788**	**.825**	**.104**	**1819**	1715	119	<.001
**PT**																						
PT-Broad	M13CHC	Mixed	ax	.922	.914	.922	.054	555780	555572	599	<.001	32226	27	<.001	**.927**	**.915**	**.927**	**.053**	**523608**	523346	572	<.001
PT-Narw.	M13CHC	Mixed	ay	.937	.925	.937	.058	212866	212748	194	<.001	44343	14	<.001	**.950**	**.936**	**.950**	**.054**	**168551**	168405	180	<.001
PT-Narw.	M13CHC	Mixed	az												.948	.934	.948	.055	174876	174732	181	<.001
PT-Narw.	M13CHC	CE	ba	.907	.890	.907	.071	313915	313799	195	<.001	68282	12	<.001	**.927**	**.908**	**.927**	**.065**	**245657**	245517	183	<.001
PT-Narw.	M13CHC	Drop	ba	.874	.856	.874	.081	422807	422705	202	<.001	53606	12	<.001	**.890**	**.867**	**.890**	**.078**	**369225**	369099	190	<.001
PT-Narw.	M13CHC	fix		.537	.468	.537	.155	1558306	1558202	201	<.001	702161	17	<.001	**.746**	**.681**	**.746**	**.120**	**856179**	856041	184	<.001
**Reyburn & Taylor (1941)**																						
R41	CHC	Baseln		.845	.775	.836	.092	474	426	31	<.001											
R41	CHC	Mixed	bb, bc	.739	.654	.730	.114	742	700	34	<.001	471	4	<.001	**.922**	**.883**	**.912**	**.067**	**279**	229	30	<.001
R41	CHC	CE	bc	.739	.645	.731	.116	742	698	33	<.001	469	4	<.001	**.922**	**.879**	**.912**	**.068**	**281**	229	29	<.001
R41	CHC	Drop	bc	.739	.664	.730	.113	741	701	35	<.001	472	4	<.001	**.922**	**.887**	**.912**	**.065**	**277**	229	31	<.001
R41	CHC	fix		.000	-2.83	-1.94	.381	7664	7624	35	<.001	3979	6	<.001	.000	**-1.20**	**-.404**	**.289**	**3697**	3645	29	<.001
**Schipolowski et al (2014)**																						
S14	S14	Baseln		.803	.757	.800	.173	4446	4384	74	<.001											
S14	S14		bd	.706	.648	.704	.208	6545	6487	76	<.001	3218	11	<.001	**.853**	**.794**	**.851**	**.159**	**3349**	3269	65	<.001
S14	CHC	Baseln		.787	.738	.785	.179	4781	4719	74	<.001											
S14	CHC	Mixed	be	.777	**.733**	.775	**.181**	4999	4941	76	<.001	516	9	<.001	**.800**	.729	**.798**	.182	**4501**	4425	67	<.001

Dataset	Model	2 Ind.		Higher-Order								Comparison			Bifactor							
			Notes	CFI	TLI	NFI	RMSEA	AIC	χ^2	*df*	*p*	$\Delta\chi^2$	*df*	*p*	CFI	TLI	NFI	RMSEA	AIC	χ^2	*df*	*p*
S14	CHC	CE	[b,f]	.687	.620	.685	.216	6970	6910	75	<.001	2568	9	<.001	**.804**	**.730**	**.802**	**.182**	**4420**	4342	66	<.001
S14	CHC	Drop		.784	**.738**	.782	**.179**	4839	4779	75	<.001	364	9	<.001	**.801**	.725	**.799**	.184	**4494**	4416	66	<.001
S14	CHC	fix		.670	.610	.668	.219	7339	7283	77	<.001	2759	10	<.001	**.796**	**.723**	**.794**	**.184**	**4600**	4524	67	<.001
**Thorndike (1921)**																						
T21	CHC	Mixed	[b,g]	.956	.940	.934	.061	138	94	33	<.001	22	6	.001	**.968**	**.946**	**.950**	**.057**	**127**	71	27	<.001
T21	CHC	CE	[b,g]	.956	.940	.934	.061	138	94	33	<.001	22	6	.001	**.968**	**.946**	**.950**	**.057**	**127**	71	27	<.001
T21	CHC	Drop		.948	.929	.926	.066	149	105	33	<.001	27	6	<.001	**.963**	**.939**	**.946**	**.061**	**133**	77	27	<.001
T21	CHC	fix		.206	-.051	.207	.254	1170	1128	34	<.001	931	7	<.001	**.877**	**.794**	**.862**	**.112**	**253**	197	27	<.001
**Thurstone**																						
T41	T41			.837	.826	.771	.052	5291	4935	1713	<.001	730	51	<.001	**.871**	**.858**	**.805**	**.046**	**4664**	4206	1662	<.001
**Woodcock-Johnson III**																						
WJIIIM	K12			.919	.909	.915	.060	11118	10942	473	<.001	1037	15	<.001	**.927**	**.916**	**.923**	**.058**	**10111**	9905	458	<.001
WJIIIM	K12NoCE	Mixed		.908	.899	.905	.063	12490	12330	481	<.001	630	10	<.001	**.913**	**.902**	**.910**	**.062**	**11880**	11700	471	<.001
WJIIIM	K12NoCE	CE		.908	.899	.905	.063	12490	12330	481	<.001	1313	20	<.001	**.918**	**.906**	**.915**	**.061**	**11217**	11017	461	<.001
WJIIIM	K12NoCE	Drop		.904	.896	.901	.064	12959	12803	483	<.001	893	20	<.001	**.911**	**.899**	**.908**	**.063**	**12106**	11910	463	<.001
WJIIIM	K12NoCE	fix		.748	.726	.745	.104	33139	32987	485	<.001	8468	22	<.001	**.813**	**.787**	**.811**	**.092**	**24715**	24519	463	<.001
WJIIIM	K12NoCE																					
**WIAT**																						
WIATM	WIAT-M	Baseln		.894	.874	.891	.112	5204	5112	144	<.001											
WIATM	WIAT-M	Mixed		.852	.825	.849	.131	7177	7087	145	<.001	3665	14	<.001	**.930**	**.908**	**.927**	**.095**	**3540**	3422	131	<.001
WIATM	WIAT-M	CE		.852	.825	.849	.131	7177	7087	145	<.001	3665	14	<.001	**.930**	**.908**	**.927**	**.095**	**3540**	3422	131	<.001
WIATM	WIAT-M	Drop		.844	.817	.841	.134	7555	7467	146	<.001	3753	14	<.001	**.923**	**.901**	**.921**	**.099**	**3830**	3714	132	<.001
WIATM	WIAT-M	fix		.813	.783	.811	.146	8972	8886	147	<.001	3820	15	<.001	**.895**	**.863**	**.892**	**.116**	**5182**	5066	132	<.001

Notes. [a]Due to a Heywood case in the higher order model, the variance of eVMF (or the variance of VMF) was fixed to .01 in both models.; [b]The bifactor model had a Heywood case for TKF, fixing only TKF to .01 allowed both models to run.; [c]Due to Heywood cases in the bifactor model that arose after fixing the variance of VMF, eWK and ePC were fixed to their error variances in both models.; [d]This unmodified bifactor model ran successfully with nothing fixed.; [e]Due to a Heywood case in the bifactor model that arose after fixing the variance of VMF, eAR was fixed to its error variances in both models.; [f]Due to a Heywood case in the higher-order model, eVMF and eTKF were fixed to .01.; [g]It was not possible to do a direct comparison of the bifactor mixed and higher-order models. The higher-order model yielded a Heywood case for eVMF and fixing VMF in the bifactor model led to a Heywood case on TFK. If both VMF and TFK were fixed to .01 in the bifactor model, then the model would no longer be bifactor.; [h]Due to a Heywood case in the bifactor model, the eAR was fixed to its error variance in both models.; [i]The higher-order model yielded a Heywood case for eVMF. Fixing VMF in the bifactor model led to a Heywood case for TKF. Both models ran when VMF (or eVMF) was freed and TKF (or eTKF) was fixed to .01.; [j]A Heywood case issue with the higher-order

model was resolved by fixing eWK to its error variance in both models.; [k]Due to a Heywood case in the higher-order model, eTKF (or the variance of TKF) was fixed to .01 in both models.; [l]A Heywood case issue with the higher-order model was resolved by fixing eTKF (or the variance of TKF) to .01 in both models.; [m]Due to Heywood in the bifactor model, case eAR was fixed to its error variance in both models.; [n]Due to a Heywood case in the higher-order model, eSpatial (or the variance of Spatial) was fixed to .01.; [o]Due to a Heywood case in the bifactor model, eRB was fixed to its error variance in both models.; [p]Due to a Heywood case in the higher-order model, eRB was fixed to its error variance in both models.; [q]Due to Heywood case in the bifactor model, eHF and eVa were fixed to their error variance in both models.; [r]A Heywood case issue originating with the bifactor model was resolved by fixing eWD to its error variance in both models.; [s]Due to a Heywood case in the bifactor model, eS was fixed to its error variance in both models.; [t]Due to a Heywood case in the bifactor model, eWD was fixed to its error variance in both models.; [u]Due to Heywood case in the bifactor model, eTest6 was fixed to its error variance in both models; [v]Due to Heywood case in the bifactor model, the variance of Gv (or eGv) was fixed to .01 in both models.; [w]Due to two Heywood cases in the bifactor model, it was not possible to run a full bifactor analysis; instead, a mixed model was run, leaving the Mechanical Operations factor as higher-order.; [x]Due to a Heywood case in the bifactor model, eLU was fixed to its error variance in both models.; [y]Due to a Heywood case in the higher-order model, eGF was fixed to .01.; [z]Due to Heywood cases in the bifactor model, eSim and eWR were fixed to their error variances in both models. This change resolved the Heywood case on eGF.; [aa]Due to an identification issue with the bifactor model, eEAS9 (Manual Speed and Accuracy) was fixed to its error variance in both models.; [ab]Due to a Heywood case in the bifactor model, eEAS4 (Visual Speed and Accuracy) was fixed to its error variances in both models; [ac]Due to a Heywood case in the higher-order model, eGS was fixed to .01 in higher-order model.; [ad]Due to a Heywood case in the bifactor model, eN was fixed to its error variance.; [ae]When run independently, both the bifactor and higher-order models had Heywood cases. Applying the fixes from both led to a Heywood case in the bifactor model for eP, which was resolved by fixing eP to its error variance in both models.; [af]Due to a Heywood case in the higher-order model, eNO was fixed to its error variance.; [ag]Due to a Heywood case in the bifactor model, eCO was fixed to its error variance.; [ah]Due to a Heywood case in the higher-order model, eNO was fixed to its error variance in both models; due to a Heywood case in the bifactor model, eCO was fixed to its error variance in both models.; [ai]A Heywood case arose for eGF in the higher-order model, fixing GF in the bifactor model led to a positive definite issue. However, fixing eP to its error variance in both models resolved the issues. ; [aj]Due to a Heywood case in the higher-order model eSL was fixed to .01.; [ak]Applying the fixes to the higher-order and bifactor models that were run independently led to a Heywood case in the bifactor model for eSLV. Fixing eSLV and eLFQ in both models (and removing the constraints on eSL and SL) allowed both models to run.; [al]Due to a Heywood case in the bifactor model, eLFQ was fixed to its error variance; [am]Due to a Heywood case in the higher-order model, eWM was fixed to .01.; [an]Due to a Heywood case in the bifactor model, eSLQ was fixed to its error variance; [ao]Fixing eWM in the bifactor model to match the higher-order model resulted in a series of additional Heywood cases; however, fixing eWMQ and eWMS to their error variances in both models and freeing eSLQ allowed both models to run successfully.; [ap]Due to a Heywood case in the bifactor model, eSLQ was fixed to its error variance in both models.; [aq]Due to a Heywood case in the higher-order model, eTest6 was fixed to its error variance.; [ar]Due to a Heywood case in the bifactor mode, eTest30 was fixed to its error variance.; [as]Due to Heywood cases in the higher-order and bifactor models, eTest 6 and eTest30 were fixed to their error variances.; [at]Due to a Heywood case in the higher-order model, eTest 6 was fixed to its error variance; due to a Heywood case in the bifactor model eTest3 was fixed to its error variance.; [au]Due to a Heywood case in the bifactor model, eTest15 was fixed to its error variance; [av]Due to Heywood cases in the higher-order and bifactor models, eTest6 and eTest15 were fixed to their error variances; [aw]Due to a Heywood case in the bifactor model, eARI was fixed to its error variance in both models.; [ax]Due to a Heywood case and empirical identification issues in the bifactor model, indicators loading on the Gv factor were treated as higher-order.; [ay]Due to a Heywood case in the higher-order model eKM was fixed to .01 in both models; due to empirical identification issues the indicators for the KM factor were left as higher-order in the mixed bifactor model.; [az]due to empirical identification issues the indicators for the KM factor were left as higher-order in the mixed bifactor model.; [ba]Many of the secondary loadings were dropped since they came from factors (having only two indicators) which were dropped.; [bb]Due to a Heywood case in the bifactor model, eGf (and the variance of Gf) was fixed to .01 in both models.; [bc]Due to Heywood cases in the bifactor model, the eV9 and eV2 were fixed to their error variances in both models.; [bd]Due to Heywood cases in the bifactor model, Test1 and eTest11 were fixed to their error variances in both models.; [be]Due to Heywood cases in the bifactor model, the variance of Grw (or eGRW) was fixed to .01 and eTest 3 was fixed to its error variance in both models.; [bf]Due to a Heywood case in the bifactor model, eTest10 was fixed to its error variance in both models.; [bg]Due to a Heywood case in the higher-order model, eF3 (or the variance of F3) was fixed to .01 in both models.

References

American Institutes for Research. (1960a). *Project TALENT, base year data, 1960: Codebook for combined classes.* Washington, DC: Author.

American Institutes for Research. (1960b). Project Talent, Base Year Data. ICPSR33341-v2. Ann Arbor, MI: Inter-university Consortium for Political and Social Research [distributor], 2013-05-23. doi:10.3886/ICPSR33341.v2. Retrieved July 8, 2013 from http://dx.doi.org/10.3886/ICPSR33341.v2

Arneson, J.J., Sackett, P.R., & Beatty, A.S. (2011). Ability-performance relationships in education and employment settings: Critical tests of the more-is-better and the good-enough hypotheses. *Psychological Science, 22*(1), 1336-1342.

Barto, E., Gonzalez, M., Alley, W., Shore, C.W., Shwartz, K.L., & Weissmuller, J.J. (2010). *ASVAB subtest and composite homogenization.* (Report No. AFCAPS-FR-2010-0019). Randolph Air Force Base, TX: Strategic Research and Assessment, Air Force Personnel Center, U.S. Air Force.

Beauducel, A., Brocke, B. & Liepmann, D. (2001). Perspectives on fluid and crystallized Intelligence: facets for verbal, numerical, and figural intelligence. *Personality and Individual Differences, 30,* 977-994.

Bennett, G.K., Seashore, H.G., & Wesman, A.G. (1990). *Differential Aptitude Tests.* (5th ed.). San Antonio, TX: Psychological Corporation.

Berger, F.R., Gupta, W.B., Berger, R.M., & Skinner, J. (1990). *Air Force Officer Qualifying Test (AFOQT) Form P: Test manual.* (Report No. AFHRL-TR-89-56). Brooks Air Force Base, TX: Air Force Human Resources Laboratory, Air Force Systems Command, U.S. Air Force.

Breaux, K.C. (2009) *Wechsler Individual Achievement Test* (3rd ed.). San Antonio, TX: Pearson.

Brown, K.G., Le, H., & Schmidt, F.L. (2006). Specific aptitude theory revisited: Is there incremental validity for training performance. *International Journal of Selection and Assessment, 14*(2), 87-100.

Campbell, E. A. (1979). *Publications based on Project TALENT Data: An annotated bibliography.* Palo Alto, CA: American Institutes for Research.

Carretta, T. R., & Ree, M. J. (1995). Near identity of cognitive structure in sex and ethnic groups. *Personality and Individual Differences*, *19*(2), 149-155.

Carretta, T.R., Retzlaff, P.D., & King, R.E. (1997). *A tale of two test batteries: A comparison of the Air Force Qualifying Test and the Multidimensional Aptitude Battery.* (Report No. AL/HR-TP-1997-0052). Mesa, AZ: Aircrew Training Research Division, Human Resources Directorate, Armstrong Laboratory, Air Force Materiel Command, U.S. Air Force.

Carroll, J. B. (1993). *Human cognitive abilities: A survey of factor analytic studies.* New York: Cambridge University Press.

Charter, R.A. (2003). A breakdown of reliability coefficients by test type and reliability method, and the clinical interpretations of low reliability. *Journal of General Psychology, 130*(3), 290-304.

Colom, R., Abad, F.J., Garcia, L.F., & Juan-Espinosa, M. (2002). Education, Wechsler's full scale IQ, and *g*. *Intelligence, 20,* 449-462.

Cureton, E. E. (1968). *A factor analysis of Project TALENT tests and four other batteries.* Washington, DC and Pittsburgh, PA: American Institutes for Research and School of Education, University of Pittsburgh.

Detterman, D.K. (1985). *A study of learning rates.* Unpublished study at Air Force Human Resources Lab.

Drasgow, F., Nye, C.D., Carretta, T.R., & Ree, M.J. (2010). Factor structure of the Air Force Officer Qualifying Test Form S: Analysis and comparison with previous forms. *Military Psychology, 22*, 68-85.

Elliott, C.D. (1990). *Differential Ability Scales: Introductory and technical handbook.* San Antonio, TX: Psychological Corporation.

Elliott, C.D. (2011). *British Ability Scales: Technical manual.* London, UK: GL Assessment Limited.

Flanagan, J.C., Dailey, J.T., Shaycroft, M.F., Gorham, W.A., Orr, D.B., & Goldberg, I. (1962). *Design for a study of American Youth.* Boston, MA: Houghton Mifflin.

Flanagan, J.C., Dailey, J.T., Shaycroft, M.F., Gorham, W.A., Orr, D.B., Goldberg, I., & Neyman, C.A., Jr. (1961). *Project TALENT.* Washington, DC: Project Talent Office, American Institutes for Research.

Flanagan, J.C., Davis, F.B., Dailey, J.T., Shaycroft, M.F., Orr, D.B., Goldberg, I., Neyman, C.A., Jr. (1964). The American high-school student: The identification, development, and utilization of human talents. Pittsburgh, PA: Project TALENT Office, University of Pittsburgh.

Guilford, J.P., & Lacey, J.I. (1947). Printed Classification Tests. Army Air Forces Aviat. Psycholol. Program Research Report No. 5. Washington, DC.: U.S. Government Printing Office.

Gustafsson, J-E., Linsdtrom, B, & Bjorck-Akesson, E. (1981). *A general model for the organization of cognitive abilities.* Unpublished study for the National Swedish Board of Education.

Hartigan, J.A & Wigdor, A.K. (1989). *Fairness in employment testing: Validity generalization, minority issues, and the General Aptitude Test Battery.* Washington, DC: National Academy Press.

Jackson, D.N. (1985). *Multidimensional Aptitude Battery.* Port Huron, MI: Research Psychologist Press.

Johnson, W., Nijenhuis, J., & Bouchard Jr, T.J. (2008). Still just 1 g: Consistent results from five test batteries. *Intelligence, 36*, 81-95.

Kass, R.A., Mitchell, K.J., Grafton, F.C., & Wing, H. (1983). Factorial validity of the Armed Services Vocational Aptitude Battery (ASVAB), Forms 8, 9, and 10L 1981 Army applicant sample. *Educational and Psychological Measurement, 43*, 1077-1087.

Kaufman, A.S., & Kaufman, N.L. (1993). *Kaufman Adolescent & Adult Intelligence Test Manual.* Circle Pines, MN: American Guidance Services, Inc.

Kaufman, A.S., & Kaufman, N.L. (2004). *Kaufman Assessment Battery for Children – Second Edition: Manual.* Circle Pines, MN: American Guidance Service Inc.

Kaufman, S. B., Reynolds, M. R., Liu, X., Kaufman, A. S., & McGrew, K. S. (2012). Are cognitive *g* and academic achievement *g* one and the same *g*? An exploration on the Woodcock–Johnson and Kaufman tests. *Intelligence, 40*(2), 123-138.

Kettner, N. (1977). *Armed Services Vocational Aptitude Battery (ASVAB Form 5): Comparison with GATB and DAT tests.* (Technical Research Report No. 77-1). Fort Sheridan, IL: U.S. Military Enlistment Processing Command, Directorate of Testing.

Kuhlman, F. (1928). The Kuhlman-Anderson intelligence tests compared with seven others. *Journal of Applied Psychology, 12*(6), 545-594.

Kuhlman, F., & Anderson, R. (1927). *Intelligence tests for ages six to maturity: Instruction manual.* Minneapolis, MN: Educational Test Bureau.

Kyllonen, P. C, and Christal, R. E. (1990). Reasoning ability is (little more than) working memory capacity?!. *Intelligence, 14*(4), 389-433.

Kyllonen, P. C. (1993). Aptitude testing inspired by information processing: a test of the four-sources model. *The Journal of General Psychology, 120*(3), 375-405.

Kyllonen, P. C., & Christal, R. E. (1989). Cognitive modeling of learning abilities: A status report of LAMP. In R. F. Dillon & J. W. Pellegrino (Eds.), *Testing: Theoretical and applied perspectives,* (pp. 146-173). New York, NY: Praeger.

Kyllonen, P.C. (1994). Cognitive abilities testing: An agenda for the 1990s. In M. S. Rumsey, C. B. Walker, & J. H. Harris (Eds.), *Personnel selection and classification.* Hillsdale, NJ: Lawrence Erlbaum.

Kyllonen, P.C., & Alluisi, E. A. (1987). Learning and forgetting facts and skills. In G. Salvendy (Ed.), *Handbook of human factors/ergonomics.* New York, NY: Wiley.

Liepmann, D., Beauducel, A., Brocke, B., & Amthauer, R. (2007). *Intelligence Structure Test-Revised*. Gottingen, Germany: Hogrefe.

Liepmann, D., Beauducel, A., Brocke, B., & Horn, S. (2001). *Intelligence Structure Test*. Oxford, UK: Hogrefe.

Lohman, D.F., & Hagen, E.P. (2002). *Cognitive Abilities Test (Form 6): Research handbook*. Itasca, IL: Riverside.

Lucas, C.M., & French, J.W. (1953). *A factorial study of experimental tests of integration, judgment, and planning.* (Research Bulletin 53-16). Princeton, NJ: Educational Testing Service.

Major, J.T., Johnson, W., & Deary, I.J. (2012). Comparing models of intelligence in Project TALENT: The VPR model fits better than the CHC and extended Gf–Gc models. *Intelligence, 40,* 543-559.

McGrew, K.S. (2009). CHC theory and the human cognitive abilities project: Standing on the shoulders of the giants of psychometric intelligence research. *Intelligence, 37,* 1-10.

McGrew, K.S., & Woodcock, R.W. (2001a). *Technical Manual – Woodcock-Johnson III.* Itasca, IL: Riverside Publishing.

McGrew, K.S., & Woodcock, R.W. (2001b). *Woodcock-Johnson III: Technical manual.* Itasca, IL: Riverside Publishing.

McGuire, C., Hindsman, E., King, F.J., & Jennings, E. (1961). Dimensions of talented behavior. *Educational and Psychological Measurement, 21*(3).

Peterson, N.G. (1993). *Review of issues associated with speededness of GATB tests.* Washington, DC: American Institutes for Research.

Psychological Corporation. (1991). *Differential Aptitude Tests for personnel and career assessment: Technical Manual.* San Antonio, TX: Author.

Ree, M. J., & Carretta, T. R. (1994). Factor analysis of the ASVAB: Confirming a Vernon-like structure. *Educational and Psychological Measurement, 54*(2), 459-463.

Ree, M.J., & Earles, J.A. (1990). *Differential validity of a differential aptitude test.* (Technical Report No. AFHRL-TR-89-59). Brooks Air Force Base, TX: Air Force Systems Command, Manpower and Personnel Division.

Ree, M.J., Mullins, C.J., Mathews, J.J., & Massey, R.H. (1982). *Armed Services Vocational Aptitude Battery: Item and factor analyses of Forms 8, 9, and 10* (Air Force Human Resources Laboratory Technical Report No. 81-55). Brooks Air Force Base, TX: Air Force Human Resources Laboratory.

Reeve, C.L. (2001). *Using latent variable analysis to explore the contribution of general and specific abilities in academic achievement: Is there more than G?* Unpublished Doctoral Dissertation. Bowling Green, OH: Bowling Green State University.

Reeve, C.L. (2004). Differential ability antecedents of general and specific dimensions of declarative knowledge: More than *g*. *Intelligence, 32*(6), 621-652.

Reeve, C.L., Meyer, R.D., & Bonaccio, S. (2006). Intelligence-personality associations reconsidered: The importance of distinguishing between general and narrow dimensions of intelligence. *Intelligence, 34,* 387-402.

Reyburn, H.A., & Taylor, J.G. (1941). Some factors of intelligence. *British Journal of Psychology, 31*, 249-261.

Roznowski, M., Dickter, D.N., Hong, S., Sawin, L.L., & Shute, V.J. (2000). Validity of measures of cognitive processes and general ability for learning and performance on highly complex computerized tutors: Is the *g* factor of intelligence even more general? *Journal of Applied Psychology, 85*(6), 940-955.

Ruch, W.W., Stang, S.W., McKillip, R.H., & Dye, D.A. (1994). *Employee Aptitude Survey: Technical Manual.* (2nd ed.). Glendale, CA: Psychological Services, Inc.

Sawin, L., Earles, J., Goff, G.N., & Chaiken, S.R. (2001). *Advanced Personnel Testing Project: Final report*. (Report No. AFRL-HE-AZ-TP-2001-0004). Mesa, AZ: Warfighter Training Research Division, Human Effectiveness Directorate, Air Force Research Laboratory, Air Force Materiel Command, U.S. Air Force.

Schipolowski, S., Wilhelm, O., & Schroeders, U. (2014). On the nature of crystallized intelligence: the relationship between verbal ability and factual knowledge. *Intelligence, 46*, 156-168.

Schmidt, F.L., & Hunter, J. (2002). History, development, evolution, and impact of validity generalization and meta-analysis methods, 1975-2001. In K.R. Murphy (Ed.). *Validity generalization: A critical review.* Mahwah, NJ: Lawrence Erlbaum Associates, Inc.

Segall, D.O., & Monzon, R.I. (1995). *Equating Forms E and F of the P&P-GATB.* Monterey, CA: Navy Personnel Research and Development Center.

TEA S.A. (1998). *Manual del WAIS III.* [Handbook for the WAIS III]. Madrid: TEA Ediciones.

Teachout, M.S., Ree, M.J., Barto, E.L., Carretta, T.R., & King, R.E. (2014). *Joint analysis of two ability tests: Two theories, one outcome*. (Technical Report No. AFRL-RH-WP-TR-2014-0014). Wright-Patterson Air Force Base, OH: U.S. Air Force.

Thorndike, R.L. (1936). Factor analysis of social and abstract intelligence. *Journal of Educational Psychology, 27*, 231-233.

Thurstone, L. L., & Thurstone, T. G. (1941). Factorial studies of intelligence. *Psychometric Monographs. 2*.

U.S. Air Force. (n.d.). *U.S. Air Force Officer Qualifying Test: Information Pamphlet.* (Form AFPT 997).

U.S. Bureau of Labor Statistics, U.S. Department of Labor. (2012a). *National Longitudinal Survey of Youth 1979 cohort, 1979-2010 (rounds 1-24)* [computer file]. Produced and distributed by the Center for Human Resource Research, The Ohio State University. Columbus, OH.

U.S. Bureau of Labor Statistics, U.S. Department of Labor. (2012b). *National Longitudinal Survey of Youth 1997 cohort, 1997-2003 (rounds 1-7)* [computer file]. Produced by the National Opinion Research Center, the University of Chicago and distributed by the Center for Human Resource Research, The Ohio State University. Columbus, OH.

U.S. Defense Manpower Data Center (2010). *Official Site of the ASVAB.* www.official-asvab.com. Retrieved May 28, 2013.

U.S. Department of Labor. (1958). *Guide to the use of General Aptitude Test Battery – Section III: Development.* Washington, DC.

Waters, S.D. (2007). *General vs. specific ability tradeoffs in personnel selection: Consequences for validity and subgroup differences.* Unpublished Doctoral Dissertation. Minneapolis, MN: University of Minnesota.

Welsh, J.R., Jr., Kucinkas, S.K., & Curran, L.T. (1990). *Armed Service Vocational Battery (ASVAB): Integrative review of validity studies.* (Technical Report No. AFHRL-TR-90-22). Brooks Air Force Base, TX: Air Force Systems Command, Manpower and Personnel Division.

Wise, L. L. & McDaniel, M. A. (1991, August). *Cognitive factors in the ASVAB and the GATB.* Paper presented at the Annual Convention of the American Psychological Association, San Francisco, CA.

Wise, L., McLaughlin, D.H., & Steel, L. (1979). *The Project TALENT data bank handbook.* Palo Alto, CA: American Institutes for Research.

Woodcock, R.W., McGrew, K.S., & Mather, N. (2001). *Woodcock-Johnson III.* Itasca, IL: Riverside Publishing.
